# Gut microbiota and metabolites signatures of clinical response in anti-PD-1/PD-L1 based immunotherapy of biliary tract cancer

**DOI:** 10.1186/s40364-024-00607-8

**Published:** 2024-06-03

**Authors:** Chengpei Zhu, Yunchao Wang, Ruijuan Zhu, Shanshan Wang, Jingnan Xue, Dongya Zhang, Zhou Lan, Chenchen Zhang, Yajun Liang, Nan Zhang, Ziyu Xun, Longhao Zhang, Cong Ning, Xu Yang, Jiashuo Chao, Junyu Long, Xiaobo Yang, Hanping Wang, Xinting Sang, Xianzhi Jiang, Haitao Zhao

**Affiliations:** 1 Department of Liver Surgery, State Key Laboratory of Complex Severe and Rare Diseases, Peking Union Medical College Hospital, Chinese Academy of Medical Sciences and Peking Union Medical College (CAMS & PUMC), No. 1 Shuaifuyuan, Wangfujing, Beijing, 100730 China; 2 Department of General Surgery Center, Clinical Center for Liver Cancer, Beijing YouAn Hospital, Capital Medical University, Beijing, China; 3 Microbiome Research Center, Moon (Guangzhou) Biotech Ltd, Guangzhou, 510535 China; 4 Organ Transplantation Center, The First Affiliated Hospital of Shandong First Medical University, Jinan, China; 5 Division of Pulmonary and Critical Care Medicine, State Key Laboratory of Complex Severe and Rare Diseases, Chinese Academy of Medical Sciences & Peking Union Medical College (CAMS & PUMC), No. 1 Shuaifuyuan, Wangfujing, Beijing, 100730 China

**Keywords:** Biliary tract cancer, Immunotherapy, Intestinal bacteria, Metabolites

## Abstract

**Background:**

Accumulating evidence suggests that the gut microbiota and metabolites can modulate tumor responses to immunotherapy; however, limited data has been reported on biliary tract cancer (BTC). This study used metagenomics and metabolomics to identify characteristics of the gut microbiome and metabolites in immunotherapy-treated BTC and their potential as prognostic and predictive biomarkers.

**Methods:**

This prospective cohort study enrolled 88 patients with BTC who received PD-1/PD-L1 inhibitors from November 2018 to May 2022. The microbiota and metabolites significantly enriched in different immunotherapy response groups were identified through metagenomics and LC-MS/MS. Associations between microbiota and metabolites, microbiota and clinical factors, and metabolites and clinical factors were explored.

**Results:**

Significantly different bacteria and their metabolites were both identified in the durable clinical benefit (DCB) and non-durable clinical benefit (NDB) groups. Of these, 20 bacteria and two metabolites were significantly associated with survival. *Alistipes* were positively correlated with survival, while *Bacilli*, *Lactobacillales*, and Pyrrolidine were negatively correlated with survival. Predictive models based on six bacteria, four metabolites, and the combination of three bacteria and two metabolites could all discriminated between patients in the DCB and NDB groups with high accuracy. Beta diversity between two groups was significantly different, and the composition varied with differences in the use of immunotherapy.

**Conclusions:**

Patients with BTC receiving immunotherapy have specific alterations in the interactions between microbiota and metabolites. These findings suggest that gut microbiota and metabolites are potential prognostic and predictive biomarkers for clinical outcomes of anti-PD-1/PD-L1-treated BTC.

**Supplementary Information:**

The online version contains supplementary material available at 10.1186/s40364-024-00607-8.

## Background

Biliary tract cancers (BTCs) are rare aggressive tumors [1]. Most patients are diagnosed with advanced disease and have a median overall survival (OS) < 1 year [2]. The use of a combination of gemcitabine and platinum-based agents is the standard chemotherapy regimen for BTC [3]. Although combination chemotherapy may be initially effective, BTC eventually becomes chemotherapy-resistant. Anti-PD-1/PD-L1 therapy is effective against various solid tumors and is considered an alternative therapy for BTC; [4–6] however, clinical trials have demonstrated limited efficacy and variable responses. Determining which patients will respond positively to anti-PD-1/PD-L1 therapy has become a major challenge.

Previous studies have shown that the gut microbiome and microbe-derived metabolites influence the anti-PD-1/PD-L1 therapy response and can be used to predict therapy outcomes in non-small cell lung cancer (NSCLC), renal cell carcinoma (RCC), and melanoma [7, 8]. Microbiome studies of oral and gut microbiota in patients with melanoma receiving anti-PD-1 therapy showed significant differences in diversity and composition between responders and non-responders [9]. Furthermore, fecal microbiota transplantation (FMT) from responsive patients into germ-free or antibiotic-treated specific pathogen-free mice can improve anti-PD-1 efficacy [9]. A recent study reported that microbial metabolites, such as short-chain fatty acids (SCFAs), influence the immune cell landscape and are associated with the anti-PD-1/PD-L1 therapy response in several solid tumors [7, 10]. However, the microbiome and microbe-derived metabolites have not been well characterized in BTC.

Previously, we analyzed the gut microbiota in patients with hepatobiliary cancer receiving anti-PD-1-based therapy and found that the enrichment of specific bacteria may be associated with the efficacy of anti-PD-1 therapy [11]. However, clinical studies have shown that the degree of malignancy and prognosis of hepatocellular carcinoma (HCC) and BTC differ substantially. Based on these findings, we prospectively and dynamically included patients with advanced BTC receiving anti-PD-1/PD-L1 therapy to further investigate the association between the gut microbiome, microbial metabolites, and clinical response (Supplementary Fig. S1).

## Methods

### Study cohort

**Fig. 1:**
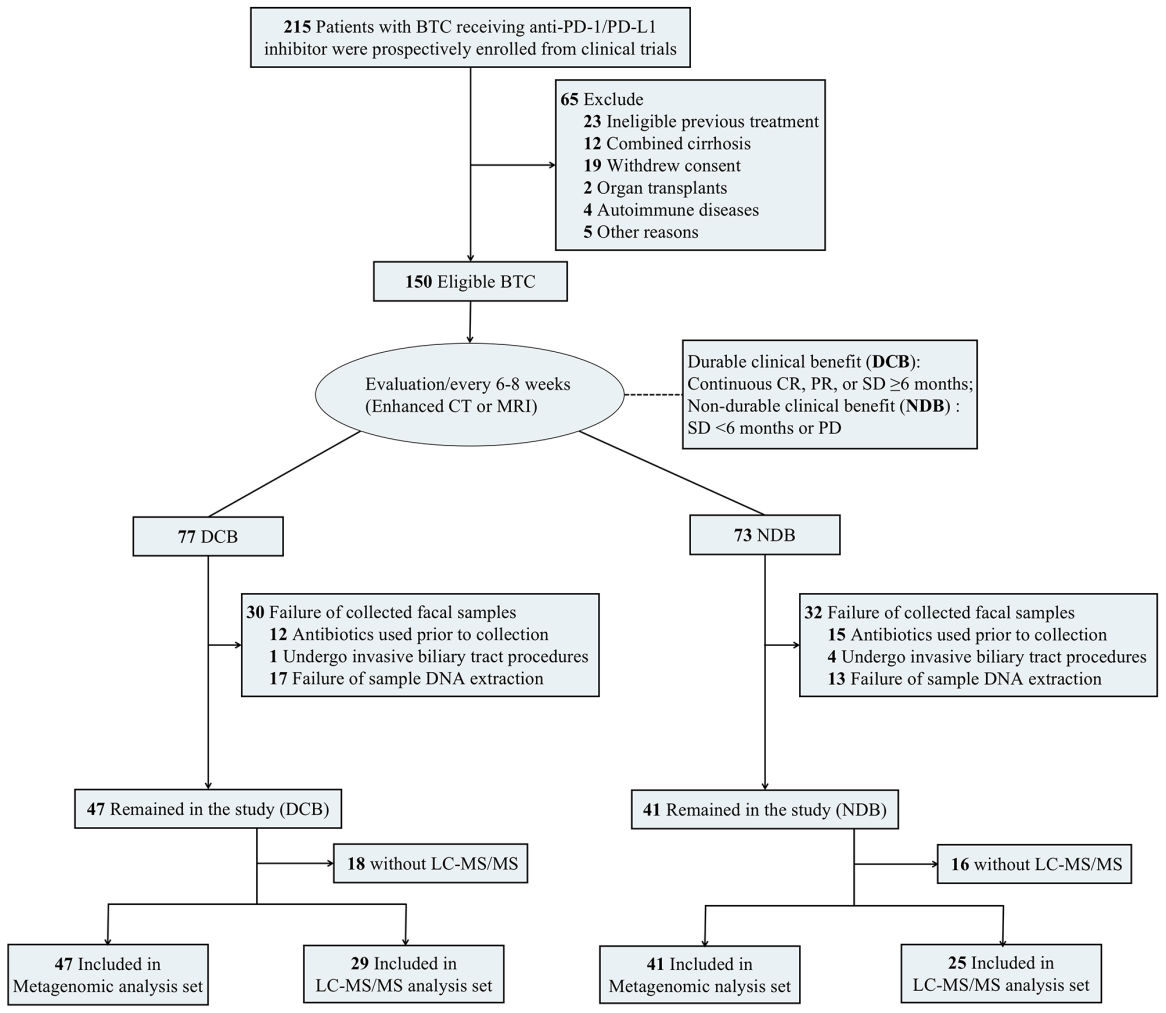
Study design

From November 2018 to May 2022, we prospectively collected fecal samples from 215 patients with advanced BTC in three clinical cohort studies (NCT03892577, NCT04010071, and NCT03895970) at Peking Union Medical College Hospital (PUMCH), as well as including our previous study [11]. Included patients met the following criteria: (i) treatment with PD-1/PD-L1 inhibitor; (ii) histologically or cytologically confirmed adenocarcinoma (extrahepatic cholangiocarcinoma (ECC), intrahepatic cholangiocarcinoma (ICC), or gallbladder cancer (GBC)); and (iii) other criteria including adequate function of major organs, an Eastern Cooperative Oncology Group (ECOG) performance status of 0–2, and radiologically-evaluable lesions. The study excluded patients who had used antibiotics, underwent invasive biliary tract procedures, used other experimental drugs within 4 weeks prior to study initiation, had undergone organ transplants, and those with combined cirrhosis or autoimmune diseases. Finally, 127 patients were excluded for the above reasons, and 88 patients were enrolled in this study (Fig. 1).

The PD-1/PD-L1 inhibitor was administered intravenously every 3 weeks at the recommended dose. Treatment was terminated when disease progression or intolerable toxicities were observed. The study was followed up until January 2023. The clinical response was assessed according to RECIST 1.1 at every 6–8 weeks [12]. The efficacy assessment included the confirmed complete response (CR), partial response (PR), stable disease (SD), and disease progression (PD). Patients with continuous CR, PR, or SD ≥ 6 months were classified into the durable clinical benefit (DCB) group, and those with SD < 6 months or PD into the non-durable clinical benefit (NDB) group [13]. Progression-free survival (PFS) was defined as the time from the start of PD-1/PD-L1 inhibitor treatment to tumor progression or death. OS was defined as the overall time from the start of the PD-1/PD-L1 inhibitor treatment until death from any cause. Fecal samples were collected before treatment, and on the day before each anti-PD-1/PD-L1 therapy with dynamic collection. All fresh fecal samples were immediately stored in sterile containers at -80 °C.

### Metagenomic sequencing analysis

Bacterial genomic DNA was extracted using the Feces Genomic DNA Purification Kit (BIOER, China). DNA concentration and purity were measured using a Qubit 3.0 (Thermo Fisher Scientific, Waltham, MA, USA) and Nanodrop One (Thermo Fisher Scientific). Sequencing libraries were generated using NEB Next® Ultra™ DNA Library Prep Kit for Illumina® (NewEngland Biolabs, MA, USA) following the manufacturer’s recommendations. The library was sequenced on an Illumina NovaSeq 6000 platform and 150-bp paired-end reads were generated. Raw data were processed using fastp (v0.19.7) to acquire clean data [14]. Trimmed reads aligned to the *Homo sapiens* genome assembly hg37 [15] were removed using KneadData integrated Bowtie (https://huttenhower.sph.harvard.edu/kneaddata/) to obtain metagenomic DNA sequences. The MetaPhlAn tool (v4.0.3) was used to quantitatively profile the taxonomic composition of the metagenome [16], whereas HUMAnN (v3.0.0. alpha.4) was used to estimate the microbial metabolic and functional pathways [17]. Functional pathway analysis included metabolic pathway (MetaCyc) and Kyoto Encyclopedia of Genes and Genomes (KEGG) functions. Alpha diversity was performed to evaluate the complexity for each sample using four indices: observed species, Simpson, Shannon, and inv. Simpson. Beta diversity calculations were applied to analyze the diversity in the samples for species complexity using principal coordinate analysis (PCoA). The *P*-value for analysis of similarities (ANOSIM) was obtained using a permutation test. Linear discriminant analysis Effect Size (LEfSe) was performed using the nonparametric factorial Kruskal-Wallis rank sum test and linear discriminant analysis (LDA) to determine the differential taxa between groups.

### Liquid chromatography (LC)-mass spectrometry (MS)/MS analysis

Briefly, fecal samples were directly added into extraction solution (methanol: acetonitrile: water = 2:2:1, containing isotope labeled internal standard mixture), shaken and mixed, and then centrifuged; the supernatant was collected and dried to obtain the metabolite extract. The samples were analyzed using an LC-MS/MS system. LC-MS/MS analyses were performed using an UHPLC (Vanquish, Thermo Fisher Scientific) with a UPLC BEH (2.1 mm×100 mm, 1.7 μm) Amide column coupled to Q Exactive HFX mass spectrometer (Orbitrap MS, Thermo). The auto-sampler temperature was 4 °C, and the injection volume was 3 µL. The QE HFX mass spectrometer was used for its ability to acquire MS/MS spectra on information-dependent acquisition mode in the control of the acquisition software (Xcalibur, Thermo). Metabolites were detected in both negative and positive ion models. The ESI source conditions were set as following: sheath gas flow rate as 30 Arb, Aux gas flow rate as 25 Arb, capillary temperature 350 °C, full MS resolution as 60,000, MS/MS resolution as 7500, collision energy as 10/30/60 in NCE mode, spray Voltage as 3.6 (positive) or-3.2 kV (negative), respectively. All procedures followed the manufacturer’s instructions.

The raw data were converted to mzXML format using ProteoWizard [18] and were preprocessed as follows: filtering a single peak to remove noise, the deviation value was filtered based on the relative standard deviation (RSD, namely coefficient of variation, CV); filtering a single peak, only the peak area data with one group of null values not more than 50% or all groups of null values not more than 50% were retained; simulate missing values in the raw data (missing value recoding), the numerical simulation method was filled by the minimum half method; data normalization, an internal standard (IS) was used for normalization.

Principal component analysis (PCA) was performed using SIMCA software. To further reveal differences between groups, orthogonal partial least squares-discriminant analysis (OPLS-DA) modeling was performed, and the quality of the model was tested by seven-fold cross validation. Student’s t test with a *P*-value less than 0.05 was used to screen differential metabolites, and the variable importance in the projection (VIP) of the first principal component of the OPLS-DA model was greater than 1. The KEGG and PubChem (https://pubchem.NDBi.nlm.nih.gov/) were used to annotate the metabolites.

### Statistical analysis

Permutational multivariate analysis of variance (PERMANOVA; permutations = 9999, R 4.2.0, vegan package [19]) was performed to investigate the effect of each clinical characteristic based on the Bray-Curtis distance matrix of the species abundance profiles of the samples. Canonical correspondence analysis (CCA) (R 4.2.0, vegan package) was used to assess the effects of each clinical characteristic based on the species abundance profile of the samples. Spearman’s correlation coefficients between differential enriched features were determined. Survival curves were estimated using the Kaplan-Meier method and compared using the log-rank test. Patients were categorized into high- and low-abundance groups based on the mean values of the relative abundances of different taxa. Survival-related taxa can be categorized as either beneficial for survival (high-abundance in DCB group, or low-abundance in NDB group) or not conducive to survival (low-abundance in DCB group, or high-abundance in NDB group). Finally, the survival benefits were categorized based on the combined expression of gut bacterial and metabolite profiles. A group exhibiting both benefits was classified as the good group, a group with no benefits was categorized as the poor group, and a group displaying one benefit and one lack of benefit was classified as the moderate group. Predictive models were built to differentiate the DCB and NDB groups based on the microbial features (species level), metabolic features, and a combination of the two types of data. The train and test sets were randomly assigned in a ratio of 2:1. Different numbers of taxa were selected to build a random forest model, key species or metabolites screened using MeanDecreaseAccuracy and MeanDecreaseGin, five-fold cross-validation, and a receiver operating characteristic (ROC) curve created to evaluate the optimal model describing the main differential species or metabolites between DCB and NDB groups by R (v4.2.0, randomForest, and pROC package), as described previously [20]. Based on the microbial model, a total of six features were selected from 60 species, while based on the metabolite model, four features were selected from 24 metabolites. Based on these six species and four metabolites, five features including three species and two metabolites were ultimately selected to construct a combined predictive model of bacteria and metabolites. Unless otherwise stated, all statistical analyses were conducted using R software (version 4.2.0), and *P* values < 0.05 were considered statistically significant.

## Results

### Patient characteristics

A total of 215 patients with BTC receiving anti-PD-1/PD-L1 inhibitor were prospectively studied, and 88 (mean [SD] age, 60.8 [10.1] years; 52 [59.1%] male) were enrolled in this study (Fig. 1). The median follow-up time was 25.1 (95% confidence interval (CI): 16.2–31.2) months. Patient characteristics of the metagenomic and LC-MS/MS analysis sets are shown in the Table 1. All patients were treated with PD-1/L1 inhibitors in combination with different types of molecular targeted therapy, and 11 patients also received chemotherapy (Supplementary Table S1). PERMANOVA analysis suggested that sex, age, and therapeutic regimen of the cohort had no significant effect on gut microbiota (Supplementary Table S2).

**Table 1:** Clinical characteristics of the study population in Metagenomic and LC-MS/MS analysis sets

	Metagenomic analysis set (*n* = 88)				LC-MS/MS analysis set (*n* = 54)		
Characteristic	No. of patients	DCB (%)	NDB (%)		No. of patients	DCB (%)	NDB (%)
Pathology							
ICC	55	23 (48.9)	32 (78.0)		34	13 (44.8)	21 (84.0)
GBC	17	13 (27.7)	4 (9.8)		11	7 (24.1)	4 (16.0)
ECC	16	11 (23.4)	5 (12.2)		9	9 (31.0)	0
Response							
Partial response	26	26 (55.3)	NA		16	16 (55.2)	NA
Stable disease	36	21 (44.7)	15 (36.6)		24	13 (44.8)	11 (44.0)
Progressive disease	26	NA	26 (63.4)		14	NA	14 (56.0)
Sex							
Female	36	23 (48.9)	13 (31.7)		20	12 (41.4)	8 (32.0)
Male	52	24 (51.1)	28 (68.3)		34	17 (58.6)	17 (68.0)
Age, y							
≥ 60	51	28 (59.6)	23 (56.1)		32	16 (55.2)	16 (64.0)
< 60	37	19 (40.4)	18 (43.9)		22	13 (44.8)	9 (36.0)
Hepatitis							
Yes	16	6 (12.8)	10 (24.4)		9	1 (3.4)	8 (32.0)
No	72	41 (87.2)	31 (75.6)		45	28 (96.6)	17 (68.0)
ECOG							
0	56	33 (70.2)	23 (56.1)		33	21 (72.4)	12 (48.0)
1	30	14 (29.8)	16 (39.0)		19	8 (27.6)	11 (44.0)
2	2	0	2 (4.9)		2	0	2 (8.0)
Child–Pugh							
A	68	38 (80.9)	30 (73.2)		41	24 (82.8)	17 (68.0)
B	20	9 (19.1)	11 (26.8)		13	5 (17.2)	8 (32.0)
Grade							
Poor	25	16 (34.0)	9 (22.0)		16	10 (34.5)	6 (24.0)
Moderate	32	18 (38.3)	14 (34.1)		21	12 (41.4)	9 (36.0)
Well	7	2 (4.3)	5 (12.2)		3	0	3 (12.0)
NA	24	11 (23.4)	13 (31.7)		14	7 (24.1)	7 (28.0)
TNM stage							
II	7	5 (10.6)	2 (4.9)		2	2 (6.9)	0
III	40	20 (42.6)	20 (48.8)		26	14 (48.3)	12 (48.0)
IV	41	22 (46.8)	19 (46.3)		26	13 (44.8)	13 (52.0)
Vascular invasion							
Yes	19	9 (19.1)	10 (24.3)		11	5 (17.2)	6 (24.0)
No	67	38 (80.9)	29 (70.7)		43	24 (82.8)	19 (76.0)
NA	2	0	2 (4.9)				
Intrahepatic metastasis							
Yes	56	31 (66.0)	25 (61.0)		41	24 (82.8)	17 (68.0)
No	31	16 (34.0)	15 (36.6)		13	5 (17.2)	8 (32.0)
NA	1	0	1 (2.4)		0	0	0
Tumor number							
1	20	13 (27.7)	7 (17.1)		4	4 (13.8)	0
2	10	3 (6.4)	7 (17.1)		5	1 (3.4)	4 (16.0)
3	2	1 (2.1)	1 (2.4)		1	0	1 (4.0)
> 3	36	23 (48.9)	13 (31.7)		26	18 (62.1)	8 (32.0)
NA	20	7 (14.9)	13 (31.7)		18	6 (20.7)	12 (48.0)
Size^#^							
≥ 5 cm	41	19 (40.4)	22 (53.7)		26	12 (41.4)	14 (56.0)
< 5 cm	47	28 (59.6)	19 (46.3)		28	17 (58.6)	11 (44.0)
Alpha fetoprotein							
≥ 20 U/mL	6	4 (8.5)	2 (4.9)		3	2 (6.9)	1 (4.0)
< 20 U/mL	79	42 (89.4)	37 (90.2)		49	26 (89.7)	23 (92.0)
NA	3	1 (2.1)	2 (4.9)		2	1 (3.4)	1 (4.0)
CA19−9							
≥ 200 U/mL	24	10 (21.3)	14 (34.1)		16	6 (20.7)	10 (40.0)
< 200 U/mL	63	37 (78.7)	26 (63.4)		38	23 (79.3)	15 (60.0)
NA	1	0	1 (2.4)		0	0	0
Total bilirubin							
≥ 17 umol/L	35	20 (42.6)	15 (36.6)		24	13 (44.8)	11 (44.0)
< 17 umol/L	53	27 (57.4)	26 (63.4)		30	16 (55.2)	14 (56.0)
Direct bilirubin							
≥ 7 umol/L	33	13 (27.7)	20 (48.8)		22	9 (31.0)	13 (52.0)
< 7 umol/L	55	34 (72.3)	21 (51.2)		32	20 (69.0)	12 (48.0)
Bile acids							
≥ 10 umol/L	35	21 (44.7)	14 (34.1)		19	10 (34.5)	9 (36.0)
< 10 umol/L	50	23 (48.9)	27 (65.9)		32	16 (55.2)	16 (64.0)
NA	3	3 (6.4)	0		3	3 (10.3)	0
Therapeutic regimen							
IM + MTT	77	40 (85.1)	37 (90.2)		48	25 (86.2)	23 (92.0)
IM + MTT + CHEMO	11	7 (14.9)	4 (9.8)		6	4 (13.8)	2 (8.0)

^#^Maximum tumor diameter. DCB, durable clinical benefit; NDB, non-durable clinical benefit; ICC, intrahepatic cholangiocarcinoma; GBC, gallbladder cancer; ECC, extrahepatic cholangiocarcinoma; ECOG, Eastern Cooperative Oncology Group; TNM, tumor node metastasis; CA19-9, Carbohydrate antigen 19 − 9; IM, immunotherapy; MTT, molecular targeted therapy; CHEMO, chemotherapy

### Integrated analysis of the microbiome and microbe-derived metabolites in anti-PD-1/PD-L1 based immunotherapy in patients with BTC

The alpha diversity was not different between DCB and NDB groups; however, the beta diversity showed significant differences between the groups (Supplementary Fig. S2). The numbers of common and unique species were 703 and 198 for DCB and NDB, respectively (Supplementary Fig. S3A). At the phylum level, the highest proportions in both DCB and NDB groups were *Firmicutes* and *Bacteroidetes*, and the *Bacteroidetes*/*Firmicutes* ratio significantly differed (Supplementary Table S3; Supplementary Fig. S3B-C). The top 20 species accounted for approximately 49.3%, and 42.6% in the DCB and NDB groups, respectively; the highest abundance was in *Escherichiacoli* (7.76%) *and Faecalibacteriumprausnitzii* (6.59%) in the DCB and NDB groups, respectively (Supplementary Table S4; Supplementary Fig. S3D). The compositions of species in each baseline sample and other level taxa are shown in Supplementary Tables S5−8 and Supplementary Fig. S3.

**Fig. 2:**
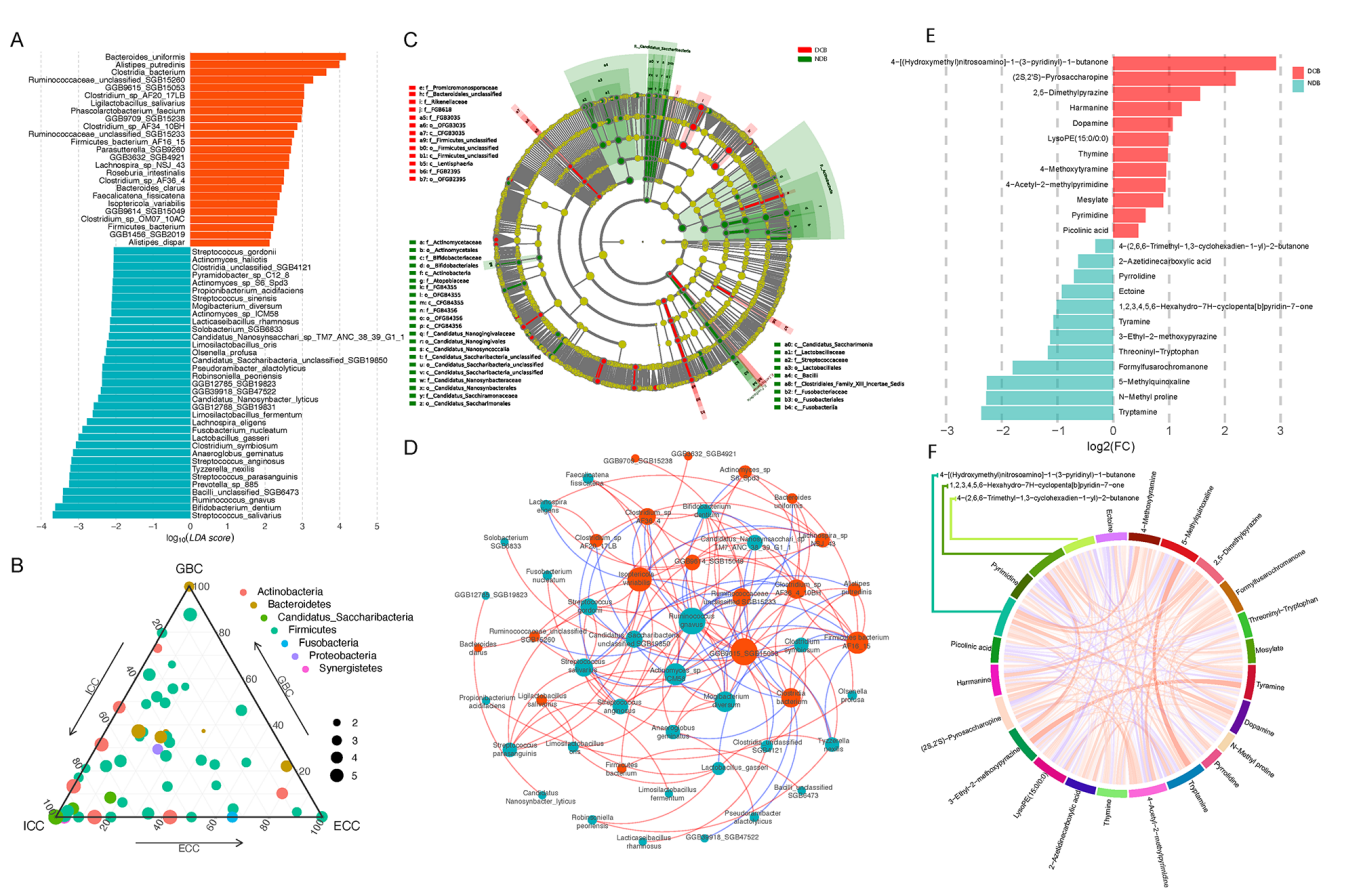
Significantly different microbiome and microbe-derived metabolites in anti-PD-1/PD-L1 based immunotherapy of BTC. (**A**) LEfSe identified significantly different abundant species in the DCB and NDB group (LDA > 2.0, *P* < 0.05). The red dots represent enrichment in the DCB group, and the cyan dots represent enrichment in the NDB group. (**B**) Ternary phase diagram for the prominent species. The different colored dots show the phylum corresponding to species. (**C**)Taxonomic cladogram from LEfSe showed different taxa enriched in the DCB and NDB groups (LDA > 2.0, *P* < 0.05). (**D**) Cooccurrence network of different species based on the Spearman correlation algorithms. The red dots represent enrichment in the DCB group, and the cyan dots represent enrichment in the NDB group. Red lines indicate positive correlations, and blue lines indicate negative correlations. (**E**) Significantly different metabolites between DCB and NDB groups. (**F**) Chord plot analysis for DCB vs. NDB comparisons using the Spearman correlation algorithms. The red connecting band indicates a positive correlation, and the blue connecting band indicates a negative correlation

We further identified 135 enriched taxa between the DCB and NDB groups, including four phyla, 11 classes, 14 orders, 21 families, 25 genera, and 60 species, using the LEfSe analysis (LDA > 2.0, *P* < 0.05) (Supplementary Fig. S4). The details of 60 species and their relative expression levels in DCB and NDB are shown in Fig. 2A and Supplementary Fig. S4. The distribution differences of species among GBC, ICC, and ECC were not obviously different, and the phylum corresponding to the differential taxa was mainly *Firmicutes* (Fig. 2B). A total of 135 different taxa showed an extremely high correlation between the different levels of taxa (Fig. 2C), and the co-expression network diagram based on species enriched in the DCB and NDB groups showed a high degree of internal interaction (Fig. 2D). The quality control of LC-MS/MS, and screening of differential metabolites are shown in Supplementary Fig. S5. Most metabolites detected were not different, while 24 were significantly different in DCB versus NDB (Fig. 2E; Supplementary Table S9). Chord plot analysis showed a higher internal correlation for 24 metabolites between DCB and NDB (Fig. 2F). Twenty-one patients (DCB, 11; NDB, 10) underwent dynamic collection of fecal samples (points 1–6). Dynamic point analyses suggest changes in species diversity and composition with the use of immunotherapy (Supplementary Tables S10-12; Supplementary Fig. S6). In DCB, the *Alistipes* tended to be stable overall, whereas in NDB, *Bifidobacterium* tended to stabilize, suggesting that the two may be involved in immunotherapy.

### Differentially enriched microbiome and microbe-derived metabolites were associated with survival benefit in anti-PD-1/PD-L1 based immunotherapy in patients with BTC

**Fig. 3:**
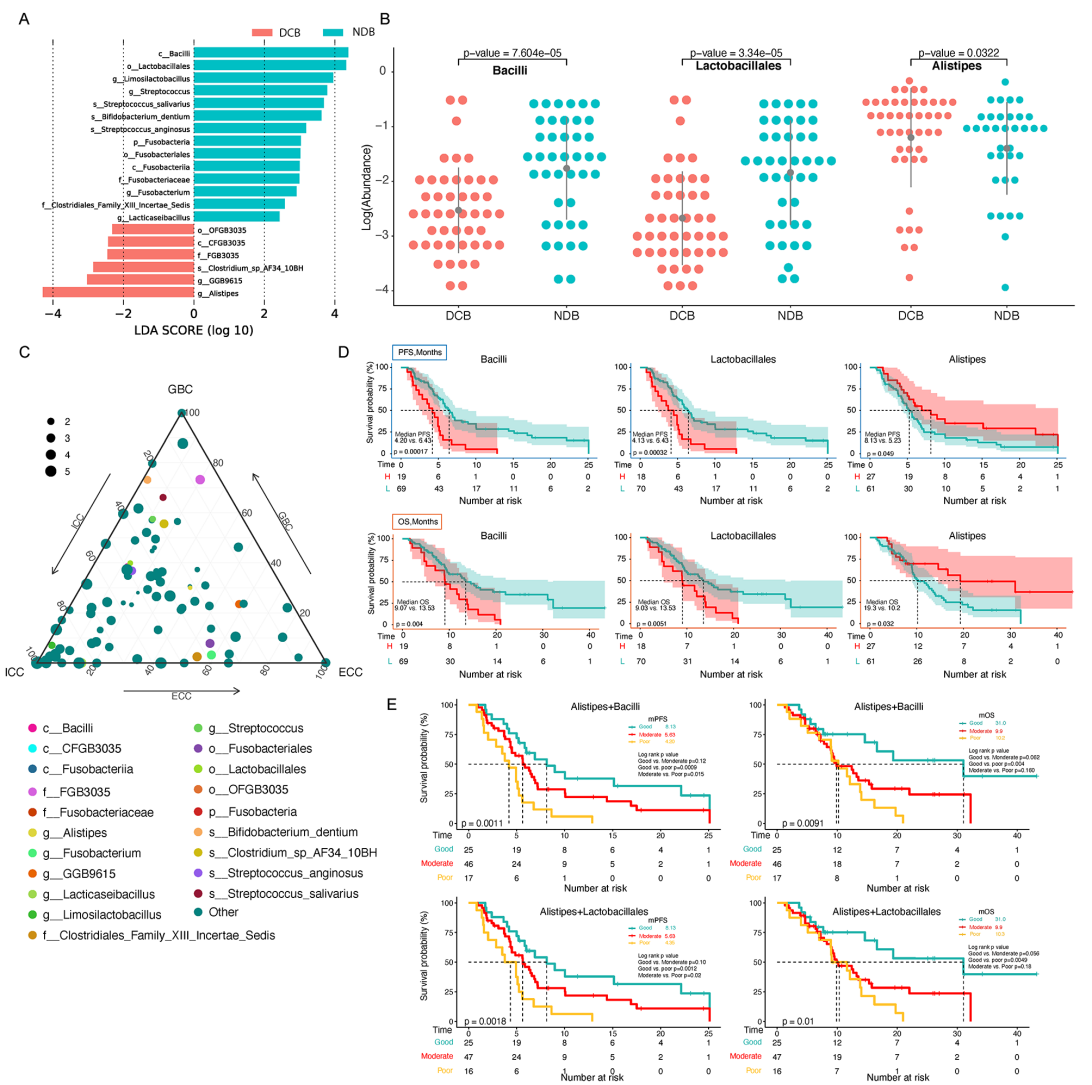
Association between gut microbiome and microbe-derived metabolites with survival. (**A**) Survival-associated significantly different taxa (*n* = 20). (**B**) Relative abundance comparison of *Bacilli*, *Lactobacillales*, and *Alistipes* between the DCB and NDB groups (Wilcoxon test). (**C**) Ternary phase diagram for the significantly different taxa. The different colored dots show survival associated taxa. (**D**) Progression-free survival (PFS) and overall survival (OS) depended on the relative abundance of *Bacilli*, *Lactobacillales*, and *Alistipes*. (**E**) PFS and OS of two combined taxa. Good signature: coexistence of enriched *Alistipes* and depleted *Bacilli*/*Lactobacillales*. Poor signature: coexistence of depleted *Alistipes* and enriched *Bacilli*/*Lactobacillales*. Moderate signature: coexistence of depleted both or enriched both two bacteria (*Alistipes* + *Bacilli*, or *Alistipes* + *Lactobacillales*)

In total, 20 taxa were statistically associated with survival benefit; the most abundant taxon were *Alistipes* and *Bacillus* in DCB and NDB, respectively (Fig. 3A; Supplementary Table S13). *Bacilli*, *Lactobacillales*, and *Alistipes* were identified as candidate biomarkers for predicted survival, using LDA > 4.0; their relative expression levels are shown in Fig. 3B. The relative proportion of *Bacilli* in the GBC subtype was high, whereas the proportion of *Alistipes* was similar in the different types (Fig. 3C).

**Fig. 4:**
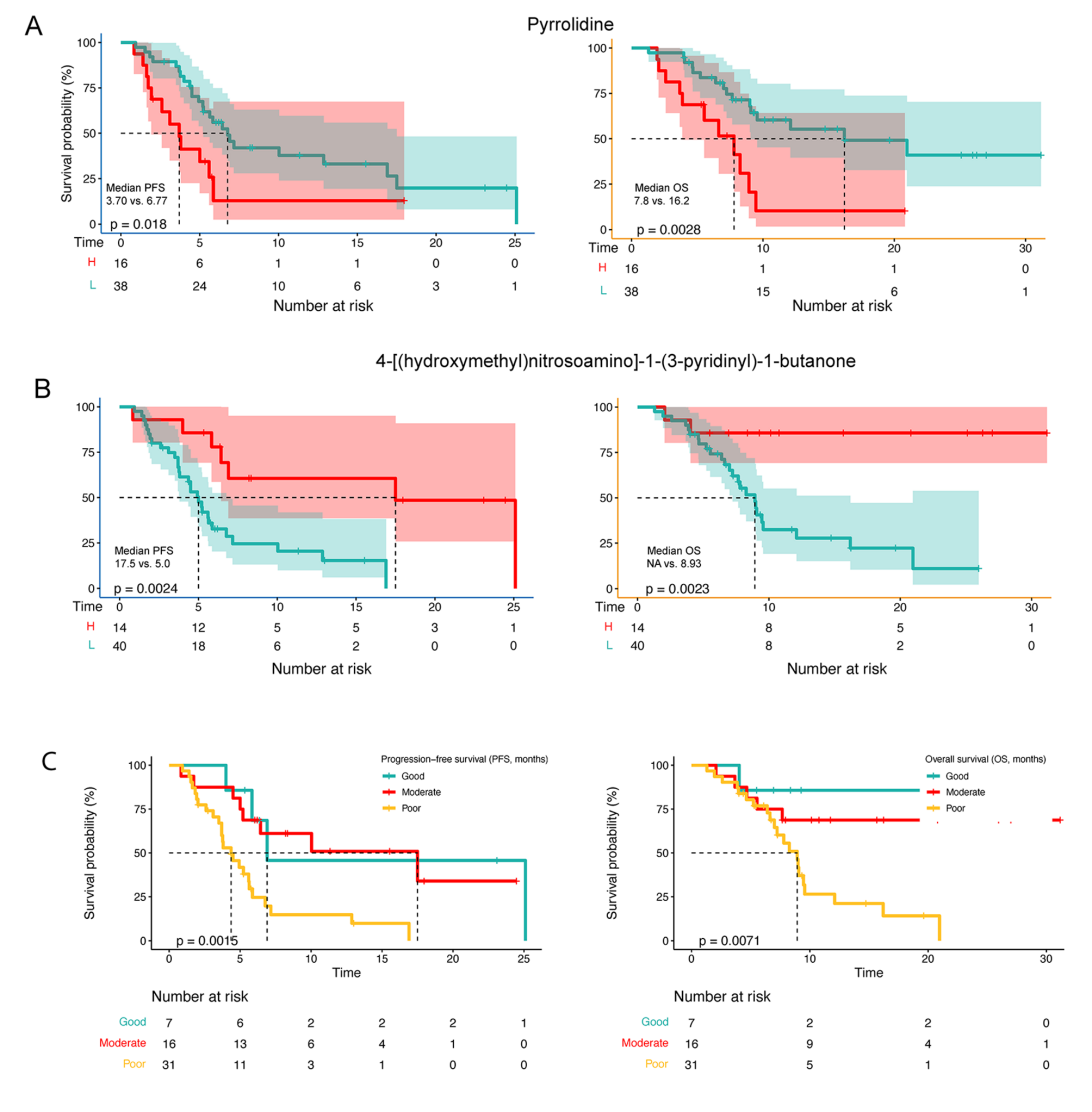
Metabolites, and combined gut microbiome and metabolites were associated with survival. (**A**) Progression-free survival (PFS) and overall survival (OS) depended on the fold change of pyrrolidine. (**B**) PFS and OS depended on the fold change of 4-[(hydroxymethyl)nitrosoamino]-1-(3-pyridinyl)-1-butanone. (**C**) PFS and OS of combined Alistipes and 4-[(hydroxymethyl)nitrosoamino]-1-(3-pyridyl)-1-butanone. High abundance of both was marked as Good group, both low marked as Low group, one high and one low, is marked as the Moderate group

Patients with enriched *Bacilli* had significantly worse PFS (median PFS: 4.20 vs. 6.43 months, *P* < 0.01) and OS (median OS: 9.07 vs. 13.53 months, *P* = 0.004; Fig. 3D). *Lactobacillales* was also associated with worse PFS (median PFS: 4.13 vs. 6.43 months, *P* < 0.01) and OS (median OS: 9.03 vs. 13.53 months, *P* = 0.005; Fig. 3D). A survival benefit was observed in patients with enriched *Alistipes* (median PFS: 8.13 vs. 5.23 months; *P* = 0.049; median OS: 19.3 vs. 10.2 months, *P* = 0.032; Fig. 3D). After combining *Alistipes* with *Bacilli* or *Lactobacillales*, differences in OS and PFS between the different expression groups were more obvious, indicating that the combination of different taxa was more reliable for predicting the effect and survival of patients with BTC on immunotherapy (Fig. 3E). Subsequently, differential metabolites were similarly analyzed for survival. Patients with low abundance of pyrrolidine benefited more a higher rate of survival (median PFS: 6.77 vs. 3.70 months, *P* = 0.018; median OS: 16.2 vs. 7.8 months, *P* = 0.0028; Fig. 4A). Patients with high abundance of 4-[(hydroxymethyl)nitrosoamino]-1-(3-pyridinyl)-1-butanone had a longer median PFS and OS compared to those low abundance (median PFS: 17.5 vs. 5.0 months, *P* = 0.0023; median OS: NA vs. 8.93 months, *P* = 0.0024; Fig. 4B). These results indicat that substance metabolism may be related to treatment efficacy and survival. In addition, we found a clear survival benefit when both taxa and metabolites beneficial for survival were present (Fig. 4C).

### Multi-omics classification for discriminating patients in the DCB and NDB group

We next assessed the potential value of using the gut microbiota and metabolites as predictive biomarkers to differentiate between DCB and NDB groups. Random forest analysis was performed based on fecal taxonomic (species abundance) or metabolic features. Model construction analyses were performed using a train set of 36 baseline samples and a test set of 18 samples, all of which were concurrently underwent metagenomic and LC-MS/MS analysis (Supplementary Table S14). We selected different numbers of taxa to build a random forest model, screened the key species or metabolics by MeanDecreaseAccuracy and MeanDecreaseGin, cross-validated the model, and drew a ROC curve to evaluate the optimal model describing the main differential species or metabolics between the DCB and NDB groups (Supplementary Table S15).

**Fig. 5:**
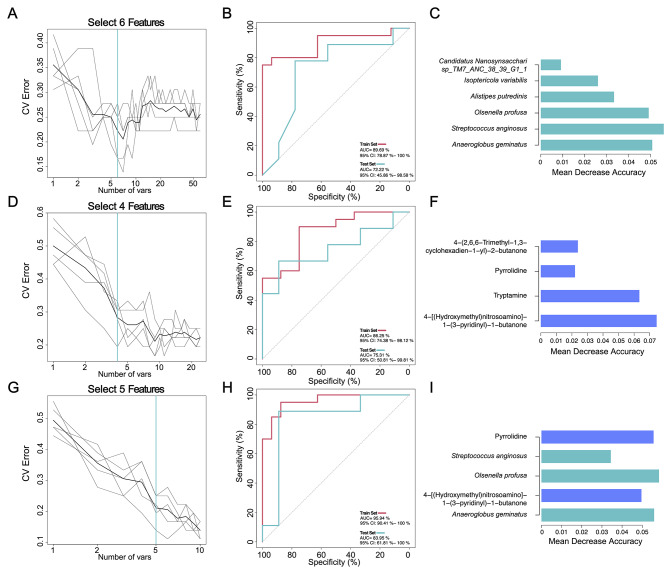
Clinical outcomes classification based on the signatures of gut microbiome and metabolome. Random forest classifiers composed of bacteria, metabolites and their combination were constructed to discriminate patients with DCB and NDB groups. The selected features (**A**), receiver operating characteristic (ROC) curve in train and test sets (**B**), and the MeanDecreaseAccuracy of selected features (**C**) based on bacteria (species level). The selected features (**D**), ROC curve in train and test sets (**E**), and the MeanDecreaseAccuracy of selected features (**F**) based on metabolites. The selected features (**G**), ROC curve in train and test sets (**H**), and the MeanDecreaseAccuracy of selected features (**I**) based on combination bacteria and metabolites

We identified a bacterial signature composed of six bacterial species that could distinguish patients with DCB and NDB (area under the curve (AUC) = 89.69% [95% CI :78.87-100%]) (Fig. 5A-C, Supplementary Table S15). In addition, the efficacy of another random forest model based on four identified fecal metabolites showed similar results (AUC = 86.25% [95% CI :74.38-98.12%]) (Fig. 5D-F, Supplementary Table S15). Notably, using three species and two metabolites, the combined model yielded an AUC of 95.94% (95% CI :90.41-100%) in discriminating between patients with DCB and NDB groups (Fig. 5G-I, Supplementary Table S15). Furthermore, we independently verified the performance of the above models in the test set independently. The microbial model could still effectively differentiate patients for DCB and NDB groups with an AUC of 72.22% (95% CI: 45.86-98.58%) (Fig. 5B), and the metabolite model with an AUC of 75.31% (95% CI: 50.81-99.81%) in the test set (Fig. 5E). Accordingly, we found that the combined marker panel could differentiate patients with DCB and NDB groups with an AUC of 83.95% (95% CI: 61.81-100%) in the test set (Fig. 5H).

### Pathways and correlations between microbes and metabolites

To investigate the mechanism through which intestinal microbes may be involved in immunotherapy, 22 KEGG pathways (LDA > 2.0, *P* < 0.05, Supplementary Table S16), 129 KO genes (Supplementary Fig. S7A), and 45 metacyclic pathways were analyzed (Supplementary Table S17). The beta diversity was statistically significant based on the KEGG and metacyclic pathways, indicating that the difference between the DCB and NDB was greater than the within-group difference (Supplementary Fig. S7B-D).

**Fig. 6:**
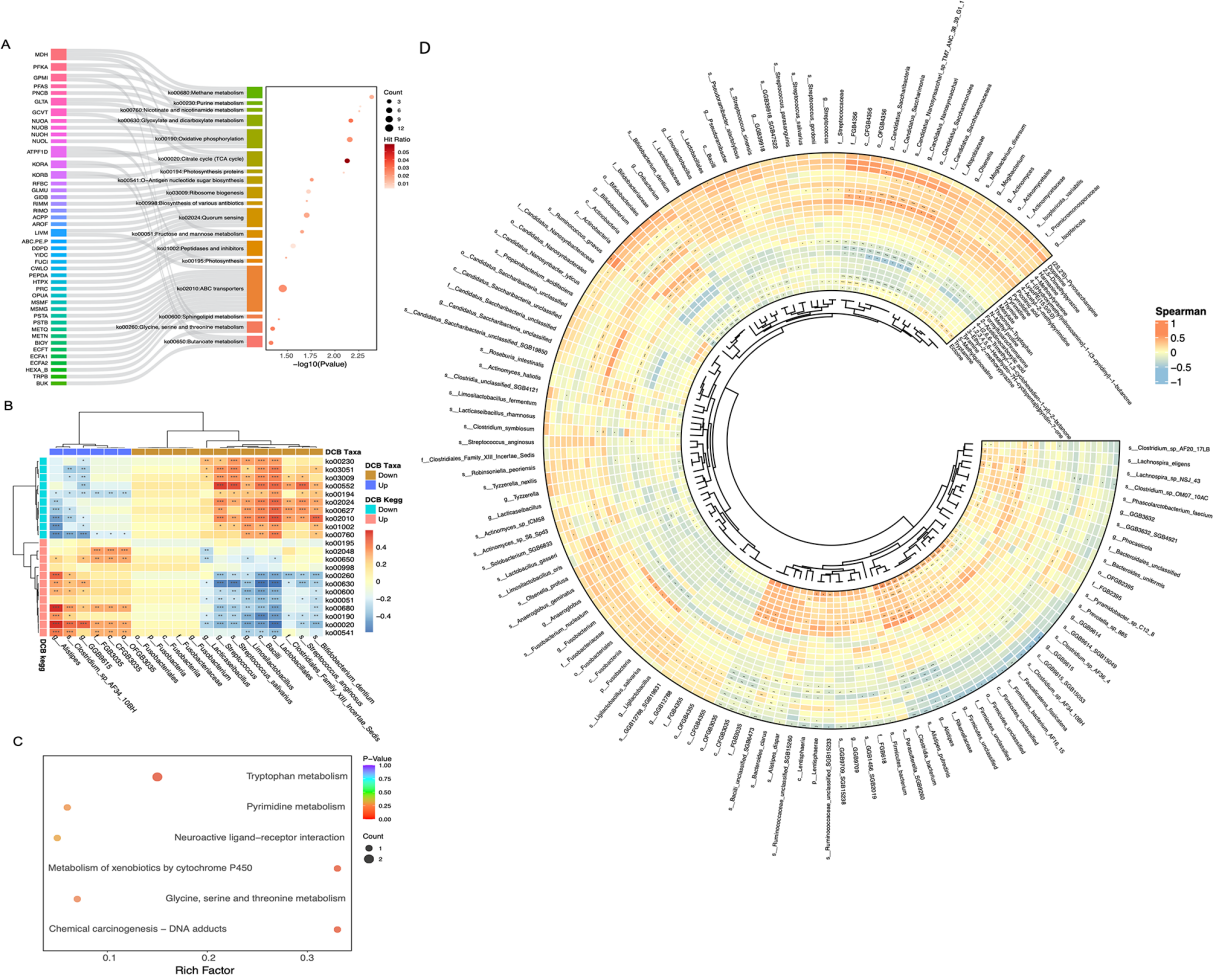
Functional annotation and correlations of significantly different gut microbiome and microbe-derived metabolites for metagenomic and LC-MS/MS analysis. (**A**) Sankey bubble plot shows differences in the Kegg pathway, ko gene, and ko gene’s contribution to the Kegg pathway. (**B**) Correlations of Kegg pathway with survival associated differentially taxa (Spearman’s rank correlation with two-tailed *P* values). (**C**) Enrichment metabolic pathways for differential metabolite annotation. (**D**) Correlations between microbes and metabolites based on differential analysis (Spearman’s rank correlation with two-tailed *P* values). *P < 0.05; **P < 0.01; ***P < 0.001

The KEGG pathway, KO gene, and KO gene contribution to the KEGG pathway were analyzed using a Sankey bubble plot (Fig. 6A). The most abundant KOs in DCB and NDB were citric acid and metabolic pathways, respectively. The DCB group was significantly enriched in energy-related pathways, including the citric acid cycle (ko0020), sphingolipid metabolism (ko00600), glyoxylate and dicarboxylate metabolism (ko00630), and fructose and mannose metabolism (ko00051) (Fig. 6A, Supplementary Table S16). The dominant metabolic pathways in DCB were related to nucleotide metabolism, whereas the NDB group was mainly composed of lipid metabolism pathways (Supplementary Table S17). The underlying mechanisms by which the gut microbiome influences the immunotherapy efficacy and survival benefits may be driven by specific bacterial species involved in different metabolic pathways.

The association analysis between KEGG pathways and differential survival taxa showed that three survival-related differential taxa were closely associated with multiple KEGG pathways, including glycine, serine, and threonine metabolism (KO00260) (Fig. 6B). Combined with the KEGG pathway database, and metabolite annotation analysis, six pathways were enriched after annotation, including KO00260 (Fig. 6C, Supplementary Table S18). The co-occurrence network of the enriched metabolic pathways is shown in Supplementary Fig. S8A. The KO00260 pathway, which co-plotted the differential KO genes and metabolites, may play an important role in microorganisms, their metabolites, and immunotherapy responses (Supplementary Fig. S8B). Most enriched taxa were associated with differential metabolites that were relatively highly expressed in the DCB group (Fig. 6D, Supplementary Fig. S8). *Alistipes* was positively correlated with metabolites mostly enriched in the DCB group, such as 4-[(hydroxymethyl)nitrosoamino]-1-(3-pyridyl)-1-butanone, and negatively correlated with metabolites enriched in the NDB group, such as pyrrolidine.

### Gut bacterial enrichment is affected by multiple clinical factors

Multiple tumor-associated factors, such as Dbil, Tbil, Child-Pugh score, Eastern Cooperative Oncology Group (ECOG) performance status, hepatitis, and vascular invasion (VI), that significantly influenced the distribution of the gut microbiome are shown by CCA (Supplementary Table S19; Supplementary Fig. S9). Hepatitis and VI correlated negatively with a favorable response, and species, such as *s__Firmicutes_bacterium* and *s__Fusobacterium_nucleatum*was, were enriched in the DCB group. Simultaneously, the effects of clinical factors on 20 survival-associated taxa were analyzed using CCA (Supplementary Table S20; Supplementary Fig. S9). We analyzed the association between survival-associated taxa and metabolites with clinical factors, and found that some clinical factors were closely associated with different species and metabolites (Supplementary Fig. S10). The heterogeneous effects of clinical factors on the intestinal microbiota indicate that the clinical response and survival benefit of immunotherapy depend on the entire gut microbiome diversity and taxonomic community richness.

## Discussion

Immunotherapy has achieved favorable results in many solid tumors; however, many tumors have demonstrated resistance to immune checkpoint inhibitor (ICI) therapy, and treatment outcomes vary greatly among patients. Evidence has shown that the gut microbiota have impact on immunotherapy. With the advancement of genomic and metabolomic technologies, the role of intestinal microbiota in tumorigenesis and treatment is gradually being recognized. However, the role and mechanisms of intestinal taxon participation remain unclear.

Evidence has emerged that intestinal microbiome can modulate the outcomes of ICI therapy through two major mechanisms: antigen-specific and antigen-independent [7]. There is an important link between gut microbiota and the immune system [21, 22]. The intestinal microbiome can not only regulate intestine local immunity, but also systemic immunity. Microbe-associated molecular patterns (MAMPs) and bacterial metabolites, such as SCFAs and bile acids, exert immunomodulatory effects on immune cells through receptors, such as TLRs and TGR5 [23]. The present study screened microbiota and metabolites that differed between the DCB and NDB groups in BTC immunotherapy, many of which can predict survival (such as *Alistipes*, *Bacilli*, and pyrrolidine) and are potential predictive biomarkers (the model combination of three bacteria and two metabolites) for predicting immunotherapy efficacy. These conclusions have also been confirmed in other cancers [24]. The microbiota was highly correlated with metabolites, suggesting that microorganisms participate in multiple immune response processes through metabolic pathways, such as the glycine, serine and threonine metabolism (KO00260 pathway).

Increased alpha diversity of the intestinal taxa in patients who respond to ICI has been previously reported [9, 25]. Studies have shown that greater baseline diversity is associated with good clinical response [26]. In this study, we observed no difference in alpha diversity; however, obvious differences in beta diversity between the DCB and NDB groups at the initiation of treatment were noted. Shaikh et al. reported that the alpha diversity of different cancers did not consistently predict the response to ICI [27]. Three studies also showed that the composition and diversity of the gut microbiota correlated with the efficacy of ICI immunotherapy [9, 28, 29]. Prospective studies have confirmed a significant association between the intestinal microflora diversity and immunotherapy efficacy in patients with NSCLC [25, 30], HCC [31], melanoma [32], and RCC [33]. Retrospective studies have suggested that in advanced solid tumors, antibiotic use may reduce the response to ICI, thereby affecting survival, and that antibiotic-induced dysbiotic intestinal flora may be causally associated with poor ICI efficacy [34, 35]. High alpha diversity with a good immune response at baseline in HCC has been reported; however, it tended to be consistent between the two groups after 6 weeks of treatment [36], which may be associated with the late development of resistance in patients who respond well to immunotherapy. The current study is the first to confirm that differences in immunotherapy efficacy and drug resistance in patients with BTC may be associated with changes in microbial diversity. This enabled us to alter the distribution of intestinal taxa in patients with BTC through oral probiotics and FMT in the future to achieve a breakthrough in treatment. Our study also found significant differences in the *Bacteroidetes*/*Firmicutes* ratio between the DCB and NDB groups, with higher ratios responding better to immunotherapy; this conclusion has been confirmed in other tumors [37, 38]. 

Moreover, *Bacteroidetes*, *Alistipes*, *Alistipes putredinis*, and *Alistipesdispar* were the main components of the DCB group in this study. Patients with high *Alistipes* expression had significantly better OS and PFS compared to those with low expression. *Alistipes putredinis* was also found to be enriched in the treatment response group in NSCLC receiving immunotherapy [25]. High *Alistipes onderdonkii* expression is associated with lasting clinical benefits in advanced NSCLC [39]. *Alistipes* may help protect against certain conditions such as liver fibrosis, colitis, cancer immunotherapy, and cardiovascular diseases [40–43]. Studies have reported a causal relationship between the increased relative abundance of *Alistipes* and decreased triglyceride concentrations [43]. Decreasing the abundance of *Alistipes* in patients with fibrotic diseases such as nonalcoholic steatohepatitis and nonalcoholic fatty liver disease leads to a decrease in SCFAs. SCFAs have anti-inflammatory effects, and *Alistipes* can produce propionic acid by expressing methylmalonyl-CoA epimerase [44]. However, other studies suggested *Alistipes* may be responsible for colorectal cancer and is associated with psychiatric symptoms of depression [45]. In mouse models of liver cancer, *Alistipes* did not only promote acetate and propionate production, but also inhibit intestinal Th17 cells, ultimately reducing the recruitment of Th17 cells to the liver, affecting the process of liver cancer [46]. Although *Alistipes* is highly expressed in patients with BTC treated with immunotherapy and can be used as a potentially better predictor of survival, further studies are needed to confirm its mechanism.

There were some limitations in this study. First, the sample size was relatively small, which may have affected the interpretation of results. No other studies on BTC immunotherapy cohorts with intestinal microbiota have been reported; therefore, larger cohorts from multiple centers, regions, and populations are needed to confirm the reliability of this study in the future. Although, additional analyses suggest there is no clear difference between cholangiocarcinoma and GBC (Supplementary Fig. S11), larger sample sizes will be needed in the future to separate and individually study the changes in intestinal microbiota and metabolites during immunotherapy for different locations of BTC. Second, the cohort design was flawed. In addition to the DCB and NDB groups, healthy population controls, metabolomics analysis of blood samples, and transcriptomics analysis of tumor tissue should be included. In addition, antibiotic consumption has been associated with poor response to immunotherapeutic PD-1 blockade in melanoma; thus, an antibiotic arm may strengthen the connection of immunotherapy to the gut microbiome in BTC. Gut fungi may also play a reciprocal role with bacteria in disease development. We performed simultaneous ITS2 sequencing of 54 samples (29 in the DCB and 25 in the NDB groups) to detect fungi, which resulted in very small differences in fungi between the two groups and fewer associations with differential bacteria and metabolites. This result suggested that fungi are relatively stable in whether BTC patients benefit from immunotherapy (Supplementary Fig. S12). Third, the results of the study are mainly based on observational, descriptive bioinformatics analyses of a research cohort, and lack in-depth biological validation and mechanistic studies. Validation experiments are needed to confirm the relationship between gut microbiota dysbiosis and the efficacy and survival benefit of anti-PD-1/PD-L1 therapy in patients. Finally, the screened prognostic and predictive markers need to be further verified in other large cohorts, and the mechanism underlying the influence of related microbiota on immunotherapy needs to be supported by further basic research.

## Conclusions

To the best of our knowledge, this study represents the first integrated multi-omics effort to explore immunotherapy-related changes in the gut microbiota and metabolites in patients with BTC. Significant differences were demonstrated in gut microbial diversity at baseline and dynamic points between the DCB and NDB groups. Patients with BTC receiving immunotherapy have specific alterations in the microbiome and metabolites interactions. Our findings suggest that the gut microbiota and metabolites have potential as prognostic and predictive biomarkers for the clinical outcomes of anti-PD-1/PD-L1-treated BTC.

### Electronic supplementary material

Below is the link to the electronic supplementary material.

**Supplementary Table S1.** Types of therapeutic regimen of the study population with 88 biliary tract cancer patients
**Supplementary Table S2.** The influence of clinical factors to gut microbiota by PERMANOVA based on species
**Supplementary Table S3.** Gut microbiome composition at the phylum level in the DCB and NDB group
**Supplementary Table S4.** Gut microbiome composition at the species level in the DCB and NDB group
**Supplementary Table S5.** Gut microbiome composition at the genus level in the DCB and NDB group
**Supplementary Table S6.** Gut microbiome composition at the class level in the DCB and NDB group
**Supplementary Table S7.** Gut microbiome composition at the order level in the DCB and NDB group
**Supplementary Table S8.** Gut microbiome composition at the family level in the DCB and NDB group
**Supplementary Table S9.** Summary of 24 significantly differential metabolites
**Supplementary Table S10.** Dynamic microbial composition in the DCB and NDB group at the species level
**Supplementary Table S11.** Dynamic microbial composition in the DCB and NDB group at the phylum level
**Supplementary Table S12.** Quantity summary of differential enriched metabolites from Point 1 to Point 6
**Supplementary Table S13.** 20 survival associated differential taxa
**Supplementary Table S14.** Clinical characteristics of the study population in train and test sets.
**Supplementary Table S15.** The performances of all prediction models by Random Forest Analysis.
**Supplementary Table S16.** Differential kegg pathways for metagenomic sequencing
**Supplementary Table S17.** Differential metacyc pathways for metagenomic sequencing
**Supplementary Table S18.** Enrichment metabolic pathways for differential metabolite annotation
**Supplementary Table S19.** The contribution of different clinical factors to the analysis of CCA based on species
**Supplementary Table S20.** The contribution of different clinical factors to the analysis of CCA based on 20 survival differential taxa
**Supplementary Fig. S1** Study workflow
**Supplementary fig. S2** The alpha diversity and beta diversity between DCB and NDB group based on species. (A) Alpha diversity. (B)Principal coordinate analysis (PCoA) of beta diversity measurements by Bray-Curtis distances. ANOSIM, *R* = 0.04, *P* = 0.025. (C)Beta diversity.
**Supplementary fig. S3** gut microbiome composition in the DCB and NDB group (A)Venn diagram of common and unique species. (B)Gut microbiome composition at the phylum level. (C)*Bacteroidetes*/*Firmicutes* ratio (B/F). (D)Gut microbiome composition at the species level. The inner circle shows the species belonging to NDB, and the outer circle shows the species belonging to DCB. (E)The composition of the top 20 species of each baseline sample. Gut microbiome composition at the genus (F), class (G), order (H), and family (I) level.
**Supplementary fig. S4** significantly different abundant taxa in the DCB group and NDB (A)All enriched taxa in DCB or NDB group. (B)Relative expression abundances in the DCB and NDB group based on species.
**Supplementary fig. S5** Differential metabolites for group DCB vs. NDB (A)Score scatter plot of PCA. (B)Score scatter plot of OPLS-DA model. (C)Permutation test of OPLS-DA model. (D)OPLS-DA S-plot. (E)Heatmap of hierarchical clustering analysis. (F)Radar chart analysis. (G)VIP scores. (H)Violin Plot of differential metabolites. (I)Z-score of differential metabolites.
**Supplementary Fig. S6** Dynamic point analysis in DCB and NDB groups. (A)Dynamic microbial composition at the species level. (B) Dynamic point alpha diversity based on species_shannon. (C) Dynamic point beta diversity based on species_Bray-Curtis distances. (D) Differential taxa and metabolites from Point 1 to Point 6. Blue font represents differential taxa, and red represents differential metabolites.
Supplementary fig. S7 functional annotation of metagenomic and metabolomics (A)Significantly different KO genes in the DCB and NDB group. The alpha diversity and beta diversity between DCB and NDB group based on Kegg pathway (B), ko gene (C), and metabolic pathway (D).
**Supplementary fig. S8** correlations between microbes and metabolites (A) Cooccurrence network of enrichment metabolic pathways by LC-MS/MS analysis. (B) KO00260 pathway co-plotted the differential KO gene and metabolites. Red indicates that the DCB group is significantly higher than the NDB group; green indicates that it is significantly lower in the DCB group than in the NDB group; pink indicates that it was higher in the DCB group than in the NDB group but not significant; bright green indicates that the DCB group is lower than the NDB group but not significant. (C)Correlations analysis of all differential species and differential metabolites.
**Supplementary fig. S9** gut microbiome distribution was affected by clinical factors Canonical correspondence analysis (CCA) with permutation test showed the clinical factors associated with the distribution of patients with BTC, the different species enriched in the DCB and NDB group, and contribution of different clinical factors to CCA for all differential taxa (A) and survival differential taxa (B). ^*^P < 0.05.
**Supplementary fig. S10** Correlations of clinical factors with differential metabolites and survival associated differentially taxa (Spearman’s rank correlation with two-tailed *P* values). *P < 0.05; **P < 0.01; ***P < 0.001.
**Supplementary Fig. S11** Comparison of intergroup differences between cholangiocarcinoma and gallbladder cancer. (A-B)Principal Coordinate analysis (PCoA) based on Bray-Curtis distances between cholangiocarcinoma (CCA) and gallbladder cancer (GBC) groups. (C) Comparison of PCoA between the four groups (CCA_DCB, CCA_NDB, GBC_DCB, and GBC_NDB).
**Supplementary Fig. S12** The differences between the DCB and NDB groups based on gut fungi. (A) alpha diversity. (B) Beta diversity. (C) Significantly different fungi. (D) Association analysis of differential fungi with differential bacteria and metabolites.

## Data Availability

Additional data from the analyses presented in this paper are available in the Supplementary material. The raw datasets used and analysed during the current study are available from the corresponding author on reasonable request. The raw sequence data reported in this paper have been deposited in the Genome Sequence Archive (Genomics, Proteomics & Bioinformatics 2021) in National Genomics Data Center (Nucleic Acids Res 2022), China National Center for Bioinformation / Beijing Institute of Genomics, Chinese Academy of Sciences (GSA-Human: HRA007409) that are publicly accessible at https://ngdc.cncb.ac.cn/gsa-human. Code for model construction uploaded to GitHub: https://github.com/zhangchenchen870529/randomForest.

## Abbreviations

BTC: biliary tract cancer
OS: overall survival
NSCLC: non-small cell lung cancer
RCC: renal cell carcinoma
FMT: Fecal microbiota transplantation
SCFAs: short-chain fatty acids
HCC: hepatocellular carcinoma
PUMCH: Peking Union Medical College Hospital
ECC: extrahepatic cholangiocarcinoma
ICC: intrahepatic cholangiocarcinoma
GBC: gallbladder cancer
ECOG: Eastern Cooperative Oncology Group
CR: confirmed complete
PR: partial response
SD: stable disease
PD: disease progression
DCB: durable clinical benefit
NDB: non-durable clinical benefit
PFS: Progression-free survival
KEGG: Kyoto Encyclopedia of Genes and Genomes
PCoA: principal coordinate analysis
ANOSIM: analysis of similarities
LEfSe: Linear discriminant analysis Effect Size
LC-MS: Liquid chromatography -mass spectrometry
LDA: linear discriminant analysis
PCA: Principal component analysis
OPLS-DA: orthogonal partial least squares-discriminant analysis
VIP: variable importance in the projection
PERMANOVA: Permutational multivariate analysis of variance
CCA: canonical correspondence analysis
ROC: reciver operating characteristic
CI: confidence interval
AUC: area under the curve
ECOG: Eastern Cooperative Oncology Group
VI: vascular invasion
ICI: immune checkpoint inhibitor
MAMPs: microbe-associated molecular patterns
