# Supplementary material for: Gut microbiota and metabolites signatures of clinical response in anti-PD-1/PD-L1 based immunotherapy of biliary tract cancer
Source: Biomark Res. 2024 Jun 3;12:56. doi: 10.1186/s40364-024-00607-8 (PMC11149318; doi:10.1186/s40364-024-00607-8)
Supplement: Supplementary file 1 — Supplementary Table S1. Types of therapeutic regimen of the study population with 88 biliary tract cancer patients Supplementary Table S2. The influence of clinical factors to gut microbiota by PERMANOVA based on species Supplementary Table S3. Gut microbiome composition at the phylum level in the DCB and NDB group Supplementary Table S4. Gut microbiome composition at the species level in the DCB and NDB group Supplementary Table S5. Gut microbiome composition at the genus level in the DCB and NDB group Supplementary Table S6. Gut microbiome composition at the class level in the DCB and NDB group Supplementary Table S7. Gut microbiome composition at the order level in the DCB and NDB group Supplementary Table S8. Gut microbiome composition at the family level in the DCB and NDB group Supplementary Table S9. Summary of 24 significantly differential metabolites Supplementary Table S10. Dynamic microbial composition in the DCB and NDB group at the species level Supplementary Table S11. Dynamic microbial composition in the DCB and NDB group at the phylum level Supplementary Table S12. Quantity summary of differential enriched metabolites from Point 1 to Point 6 Supplementary Table S13. 20 survival associated differential taxa Supplementary Table S14. Clinical characteristics of the study population in train and test sets. Supplementary Table S15. The performances of all prediction models by Random Forest Analysis. Supplementary Table S16. Differential kegg pathways for metagenomic sequencing Supplementary Table S17. Differential metacyc pathways for metagenomic sequencing Supplementary Table S18. Enrichment metabolic pathways for differential metabolite annotation Supplementary Table S19. The contribution of different clinical factors to the analysis of CCA based on species Supplementary Table S20. The contribution of different clinical factors to the analysis of CCA based on 20 survival differential taxa Supplementary Fig. S1 Study workflow Supplementary fig. S2 The al [file 40364_2024_607_MOESM1_ESM.pdf]

Supplemental material

Gut microbiota and metabolites signatures of clinical response in anti-PD-1/PD-L1 based immunotherapy of biliary tract cancer

Chengpei Zhu[1, 2, †]; YunchaoWang[1, 4, †]; Ruijuan Zhu[3, †]; Shanshan Wang[1, †]; Jingnan Xue[1]; Dongya Zhang[3]; Zhou Lan[3]; Chenchen Zhang[3]; Yajun Liang[3]; Nan Zhang[1]; Ziyu Xun[1]; Longhao Zhang[1]; Cong Ning[1]; Xu Yang[1]; Jiashuo Chao[1]; Junyu Long[1]; Xiaobo Yang[1]; Hanping Wang[1, 5, *]; Xinting Sang[1, *]; Xianzhi Jiang[3, *]; Haitao Zhao[1, *]

[1]Department of Liver Surgery, State Key Laboratory of Complex Severe and Rare Diseases, Peking Union Medical College Hospital, Chinese Academy of Medical Sciences and Peking Union Medical College (CAMS & PUMC), Beijing, China.

[2]Department of General Surgery Center, Beijing YouAn Hospital, Clinical Center for Liver Cancer, Capital Medical University, Beijing, China.

[3]Microbiome Research Center, Moon (Guangzhou) Biotech Ltd., Guangzhou, China.

[4]Organ Transplantation Center, The First Affiliated Hospital of Shandong First Medical University, Jinan, China.

[5]Division of Pulmonary and Critical Care Medicine, State Key Laboratory of Complex Severe and Rare Diseases, Chinese Academy of Medical Sciences & Peking Union Medical College (CAMS & PUMC), Beijing, China.

[†]These authors contributed equally.

*Corresponding author

Haitao Zhao, Department of Liver Surgery, State Key Laboratory of Complex Severe and Rare Diseases, Peking Union Medical College Hospital, Chinese Academy of Medical Sciences and Peking Union Medical College (CAMS & PUMC), No. 1 Shuaifuyuan, Wangfujing, Beijing 100730, China. Email: zhaoht@pumch.cn. Tel.: +86 10 69156042.

Xianzhi Jiang, Microbiome Research Center, Moon (Guangzhou) Biotech Ltd., Guangzhou 510535, China. Email: jxz@moonbio.com. Tel.: +86 20 31603387.

Xinting Sang, Department of Liver Surgery, State Key Laboratory of Complex Severe and Rare Diseases, Peking Union Medical College Hospital, Chinese Academy of Medical Sciences and Peking Union Medical College (CAMS & PUMC), No. 1 Shuaifuyuan, Wangfujing, Beijing 100730, China. Email: sangxt@pumch.cn. Tel.: +86 10 69156042.

Hanping Wang, Division of Pulmonary and Critical Care Medicine, State Key Laboratory of Complex Severe and Rare Diseases, Chinese Academy of Medical Sciences & Peking Union Medical College (CAMS & PUMC), No. 1 Shuaifuyuan, Wangfujing, Beijing 100730, China. Email: wanghp@pumch.cn. Tel.: +86 10 69156042.

Supplementary Table S1. Types of therapeutic regimen of the study population with 88 biliary tract cancer patients

Types of IM	DCB	NDB	Types of MTT	DCB	NDB	Types of CHEMO	DCB	NDB
Toripalimab	32	22	Lenvatinib	34	27	Gemox	3	2
Pembrolizumab	8	7	Axitinib	5	9	GC	3	1
Sintilimab	3	4	Apatinib	5	5	GS	1	1
Camrelizumab	1	3	Bevacizumab	1	0			
Tislelizumab	0	3	Olaparib	1	0			
Nivolumab	1	0	Surufatinib	1	0			
Durvalumab	2	1						
Envafolimab	0	1						

DCB, durable clinical benefit; NDB, non-durable clinical benefit; IM, immunotherapy; MTT, molecular targeted therapy; CHEMO, chemotherapy; Gemox, Gemcitabine plus oxaliplatin; GC, Gemcitabine plus cisplatin; GS, Gemcitabine plus S-1.

Supplementary Table S2. The influence of clinical factors to gut microbiota by PERMANOVA based on species

phenotype	SumsOfSqs	MeanSqs	F.Model	R2	Pr(>F)
Tsize[#]	0.902686464	0.902686464	2.542499868	0.028715022	1.00E-04
Dbil	0.739285599	0.739285599	2.071182185	0.023517138	0.0013
Tbil	0.713411566	0.713411566	1.997010243	0.022694069	0.0024
Sex	0.298741589	0.298741589	0.8251127	0.009503157	0.7497
Age	0.289102467	0.289102467	0.798242707	0.009196531	0.7875
TNM	0.664180369	0.332090184	0.917320895	0.021127994	0.6581
Response	0.605168254	0.302584127	0.834217559	0.019250782	0.8375
Child Pugh	0.380577192	0.380577192	1.053909371	0.012106399	0.3597
Ecog	0.80678245	0.403391225	1.119460997	0.025664256	0.2182
Hepatitis	0.377253127	0.377253127	1.044592428	0.012000658	0.3787
CA199	0.345265073	0.345265073	0.95503572	0.010983098	0.5392
Intrahepatic metastasis	0.480265506	0.480265506	1.334253146	0.015277547	0.1006
Vascular invasion	0.427901005	0.427901005	1.186768796	0.013611799	0.2041
Bile acids	0.388612001	0.388612001	1.076438183	0.012361991	0.3321
AFP	0.339842198	0.339842198	0.939871603	0.010810593	0.581
Therapeutic regimen	0.348631243	0.348631243	0.964451282	0.011090178	0.501

[#]Maximum tumor diameter.

Supplementary Table S3. Gut microbiome composition at the phylum level in the DCB and NDB group

Taxonomy	DCB	NDB
p__Firmicutes	0.432076704	0.520081905
p__Bacteroidetes	0.345363002	0.251612927
p__Proteobacteria	0.126575262	0.082768641
p__Actinobacteria	0.050019664	0.1046037
p__Verrucomicrobia	0.042843098	0.028175393
p__Bacteria_unclassified	2.05E-05	0.007023163
p__Synergistetes	0.002520647	0.002644073
p__Fusobacteria	9.74E-05	0.001921056
p__Candidatus_Saccharibacteria	0.000166168	0.001111902
p__Euryarchaeota	0.000152681	5.07E-05
p__Lentisphaerae	0.000131419	5.33E-06
p__Ascomycota	2.74E-05	0
p__Candidatus_Melainabacteria	4.75E-06	0
p__Coprothermobacterota	1.26E-06	0
p__Tenericutes	0	1.20E-06
others	8.51E-09	5.42E-18

Supplementary Table S4. Gut microbiome composition at the species level in the DCB and NDB group

Taxonomy	DCB	NDB
s__Faecalibacterium_prausnitzii	6.15%	6.59%
s__Escherichia_coli	7.76%	4.14%
s__Bacteroides_uniformis	5.70%	2.89%
s__Akkermansia_muciniphila	4.27%	2.82%
s__Phocaeicola_vulgatus	3.93%	2.84%
s__Klebsiella_pneumoniae	2.70%	2.03%
s__Alistipes_putredinis	3.16%	1.44%
s__Bifidobacterium_longum	0.82%	3.18%
s__Alistipes_onderdonkii	2.31%	1.57%
s__Bifidobacterium_adolescentis	0.70%	2.45%
s__Bacteroides_thetaiotaomicron	1.53%	1.55%
s__Gemmiger_formicilis	1.05%	1.67%
s__Streptococcus_salivarius	1.09%	1.48%
s__Parabacteroides_distasonis	0.76%	1.77%
s__Bifidobacterium_pseudocatenulatum	1.21%	1.25%
s__Lachnospiraceae_bacterium	1.09%	1.18%
s__Fusicatenibacter_saccharivorans	1.00%	1.26%
s__Clostridia_bacterium	1.34%	0.89%
s__Alistipes_shahii	1.54%	0.67%
s__Blautia_wexlerae	1.18%	1.01%
others	50.69%	57.35%

Supplementary Table S5. Gut microbiome composition at the genus level in the DCB and NDB group

Taxonomy	DCB	NDB
g__Bacteroides	0.125536177	0.094814824
g__Faecalibacterium	0.073911717	0.076038117
g__Alistipes	0.086952694	0.046217273
g__Bifidobacterium	0.036011857	0.08480621
g__Escherichia	0.077634753	0.041351288
g__Phocaeicola	0.072578891	0.037255766
g__Akkermansia	0.042742091	0.028172415
g__Prevotella	0.029565219	0.038994032
g__Klebsiella	0.029520696	0.023315166
g__Blautia	0.023673466	0.023325332
g__Ruminococcaceae_unclassified	0.02515784	0.020887822
g__Lachnospiraceae_unclassified	0.021461421	0.020081276
g__Streptococcus	0.014259055	0.025542085
g__Clostridia_unclassified	0.017642172	0.019299578
g__Parabacteroides	0.011124081	0.023816254
g__Dialister	0.008743596	0.02380158
g__Gemmiger	0.012928864	0.018651473
g__Roseburia	0.017987883	0.013477876
g__Ruminococcus	0.017537264	0.012476839
g__Veillonella	0.01362396	0.011346351
others	0.241406302	0.316328444

Supplementary Table S6. Gut microbiome composition at the class level in the DCB and NDB group

Taxonomy	DCB	NDB
c__Clostridia	0.368674198	0.383514929
c__Bacteroidia	0.344544055	0.251105027
c__Gammaproteobacteria	0.113991119	0.067408
c__Actinobacteria	0.038948111	0.088132963
c__Negativicutes	0.035788228	0.060328688
c__Bacilli	0.020364704	0.067864024
c__Verrucomicrobiae	0.042742091	0.028172415
c__Coriobacteriia	0.01105846	0.016467471
c__Betaproteobacteria	0.008132068	0.009812266
c__Deltaproteobacteria	0.004089538	0.004995915
c__Bacteria_unclassified	2.05E-05	0.007023163
c__CFGB1340	0.002918581	0.003137671
c__Synergistia	0.002520647	0.002644073
c__Erysipelotrichia	0.001185026	0.003657461
c__Fusobacteriia	9.74E-05	0.001921056
c__Firmicutes_unclassified	0.001177991	0.000143576
c__CFGB540	0.000732551	0
c__CFGB3035	0.000570053	0.000127124
c__CFGB3053	0.000389662	0.000300444
c__CFGB42691	0.00020876	0.000390717
others	0.001846202	0.002853017

Supplementary Table S7. Gut microbiome composition at the order level in the DCB and NDB group

Taxonomy	DCB	NDB
o__Clostridiales	0.337162453	0.349073237
o__Bacteroidales	0.344536043	0.251044241
o__Enterobacterales	0.109863645	0.066249117
o__Bifidobacteriales	0.036055768	0.084991846
o__Veillonellales	0.027751349	0.054705727
o__Lactobacillales	0.019692304	0.061734366
o__Verrucomicrobiales	0.042742091	0.028172415
o__Clostridia_unclassified	0.031508194	0.034441593
o__Burkholderiales	0.008031789	0.009789859
o__Coriobacteriales	0.006438821	0.009416417
o__Acidaminococcales	0.007508321	0.004390583
o__Eggerthellales	0.004567198	0.007041076
o__Desulfovibrionales	0.004089538	0.004995915
o__Bacteria_unclassified	2.05E-05	0.007023163
o__Bacilli_unclassified	0.000667181	0.006088656
o__OFGB1340	0.002918581	0.003137671
o__Synergistales	0.002520647	0.002644073
o__Erysipelotrichales	0.001185026	0.003657461
o__Pasteurellales	0.003262009	0.000705585
o__Micrococcales	0.00188803	0.001057668
others	0.007590485	0.009639332

Supplementary Table S8. Gut microbiome composition at the family level in the DCB and NDB group

Taxonomy	DCB	NDB
f__Ruminococcaceae	0.185450219	0.179243429
f__Lachnospiraceae	0.11486557	0.131643707
f__Bacteroidaceae	0.126096662	0.09589199
f__Enterobacteriaceae	0.109762747	0.065894034
f__Rikenellaceae	0.087789464	0.046375851
f__Bifidobacteriaceae	0.036055768	0.084991846
f__Bacteroidales_unclassified	0.07978326	0.038721793
f__Veillonellaceae	0.027751349	0.054705727
f__Prevotellaceae	0.03361224	0.040890395
f__Akkermansiaceae	0.042742091	0.028172415
f__Clostridia_unclassified	0.031508194	0.034441593
f__Clostridiaceae	0.021427472	0.020394122
f__Streptococcaceae	0.014261613	0.026108995
f__Tannerellaceae	0.011129991	0.023817193
f__Lactobacillaceae	0.004835028	0.029694034
f__Oscillospiraceae	0.01066176	0.009400473
f__Coriobacteriaceae	0.006315132	0.008286276
f__Sutterellaceae	0.007221645	0.007002561
f__Acidaminococcaceae	0.007508321	0.004390583
f__Eggerthellaceae	0.004567198	0.007041076
others	0.036654277	0.062891907

Supplementary Table S9. Summary of 24 significantly differential metabolites

id	Name	Mean.DCB	Mean.NDB	VIP	P.value	FDR	Fold.Change	Log2.Fold.Change
6	Mesylate	2.633	1.419	1.775	0.030	0.905	1.856	0.892
194	Tyramine	0.004	0.008	2.013	0.043	0.905	0.476	-1.071
242	Dopamine	0.184	0.088	1.024	0.047	0.905	2.089	1.063
312	N-Methyl proline	0.030	0.144	1.482	0.020	0.905	0.206	-2.277
379	Pyrrolidine	0.103	0.168	2.615	0.010	0.905	0.615	-0.701
394	Tryptamine	0.060	0.308	2.690	0.029	0.905	0.194	-2.367
441	4-Acetyl-2-methylpyrimidine	0.024	0.012	1.130	0.048	0.905	1.915	0.937
463	Thymine	0.046	0.023	2.074	0.024	0.905	1.966	0.975
491	2-Azetidinecarboxylic acid	0.039	0.060	1.940	0.035	0.905	0.649	-0.625
492	LysoPE(15:0/0:0)	1.269	0.640	1.984	0.027	0.905	1.982	0.987
575	3-Ethyl-2-methoxypyrazine	0.052	0.114	2.297	0.035	0.905	0.456	-1.134
620	(2S,2'S)-Pyrosaccharopine	0.109	0.024	1.817	0.038	0.905	4.568	2.192
627	Harmanine	0.121	0.052	1.753	0.035	0.905	2.335	1.223
670	Picolinic acid	0.016	0.012	2.446	0.030	0.905	1.363	0.447
675	4-((Hydroxymethyl)nitrosoamino)-1-(3-pyridinyl)-1-butanone	0.093	0.012	2.505	0.004	0.905	7.544	2.915
799	Pyrimidine	0.051	0.034	2.735	0.021	0.905	1.491	0.577
826	1,2,3,4,5,6-Hexahydro-7H-cyclopenta(b)pyridin-7-one	0.002	0.004	2.362	0.026	0.905	0.495	-1.015
864	4-(2,6,6-Trimethyl-1,3-cyclohexadien-1-yl)-2-butanone	0.045	0.056	2.209	0.034	0.905	0.800	-0.322
874	Ectoine	0.088	0.167	1.472	0.026	0.905	0.527	-0.923
929	4-Methoxytyramine	0.024	0.012	1.644	0.032	0.905	1.925	0.945
972	5-Methylquinoxaline	0.012	0.058	2.502	0.028	0.905	0.208	-2.264
1013	2,5-Dimethylpyrazine	0.038	0.013	2.371	0.009	0.905	2.940	1.556
1040	Formylfusarochromanone	0.076	0.267	2.167	0.022	0.905	0.286	-1.805
1044	Threoninyl-Tryptophan	0.010	0.023	2.593	0.003	0.905	0.444	-1.171

Supplementary Table S10. Dynamic microbial composition in the DCB and NDB group at the species level

	DCB_P1	DCB_P2	DCB_P3	DCB_P4	DCB_P5	DCB_P6	NDB_P1	NDB_P2	NDB_P3	NDB_P4	NDB_P5	NDB_P6
s__Faecalibacterium_prausnitzii	0.061969717	0.050899914	0.132061525	0.109846233	0.06170045	0.142921143	0.079499083	0.07447635	0.06134782	0.020588	0.0187911	0.001900433
s__Klebsiella_pneumoniae	0.008459117	0.016677943	0.000130525	0.03209185	0.066217075	0.012007029	0.0019175	0.052520933	0.00134888	0.0018536	0.074707567	0.3150445
s__Akkermansia_muciniphila	0.003479333	0.066550929	0.153100775	0.02841735	0.018892325	0.01615	0.067272967	0.03805905	0.00883916	0.007215625	0	0.167902733
s__Bacteroides_uniformis	0.051280133	0.073445429	0.189379675	0.0460699	0.0634861	0.023648343	0.038167717	0.023681683	0.0319387	0.001394325	0.007965033	0.015354267
s__bacterium_OL_1	0	0	3.00E-07	6.08E-06	0	4.71E-05	0.001505567	0.04633425	0.145862	0.1580957	0.1393175	0
s__Citrobacter_freundii	2.73E-05	5.34E-05	8.05E-06	2.63E-05	2.99E-05	7.76E-06	0.000158717	0.0004387	9.28E-06	1.00E-05	0.2784671	0.186463567
s__Escherichia_coli	0.036553883	0.101040814	0.000857725	0.083286117	0.035528275	0.013188386	0.044895533	0.010744183	0.00668624	0.016589725	0.0269629	0.016367467
s__Alistipes_onderdonkii	0.016560033	0.124025614	0.026781475	0.090431383	0.0338129	0.026245929	0.026285017	0.018459233	0.00889428	0.0034005	2.88E-05	0
s__Parabacteroides_distasonis	0.006861383	0.005818471	0.003370025	0.007826317	0.00325965	0.001134914	0.012976317	0.083261367	0.06310392	0.062702525	0.052150867	0.014450367
s__Bifidobacterium_adolescentis	0.031365567	0.000641929	0.009872525	0.019834417	0.000631175	0.004451857	0.003644883	0.06453535	0.06232776	0.085450825	0.005476633	1.54E-05
others	0.783443517	0.560845543	0.4844374	0.5821641	0.71644215	0.7601975	0.7236767	0.5874889	0.60964196	0.642699175	0.396132467	0.2825013

Supplementary Table S11. Dynamic microbial composition in the DCB and NDB group at the phylum level

	DCB_P1	DCB_P2	DCB_P3	DCB_P4	DCB_P5	DCB_P6	NDB_P1	NDB_P2	NDB_P3	NDB_P4	NDB_P5	NDB_P6
p__Firmicutes	0.588030883	0.344913	0.3934753	0.44056535	0.416770625	0.6549675	0.5767941	0.464541833	0.35868348	0.298658125	0.136947	0.169298733
p__Bacteroidetes	0.285860633	0.439311771	0.408611275	0.354057283	0.335930275	0.219435814	0.24140655	0.285221233	0.2840701	0.3427092	0.130492567	0.060463367
p__Proteobacteria	0.055573267	0.125876286	0.016085	0.12198635	0.140069225	0.039608029	0.076145117	0.078476833	0.1150367	0.029686525	0.408640933	0.587058767
p__Actinobacteria	0.066682283	0.022226614	0.02872735	0.046309317	0.0881666	0.0690537	0.027285667	0.086174233	0.08324964	0.1589978	0.184473067	0.013018433
p__Verrucomicrobia	0.003479333	0.066550929	0.153100775	0.02841735	0.018892325	0.01615	0.067272967	0.03805905	0.00883916	0.007215625	0	0.167902733
p__Bacteria_unclassified	0	0	3.00E-07	6.08E-06	0	4.71E-05	0.001505567	0.04633425	0.145862	0.1580957	0.1393175	0
p__Synergistetes	0	0.000971543	0	0.0086576	8.37E-05	0.000281929	0.0050515	0.000640783	0.00392816	0.0004698	9.77E-06	0.002216333
p__Fusobacteria	0	7.74E-06	0	0	0	5.17E-06	0.00380275	0.000169033	0.00025154	0.0033024	0	0
p__Candidatus_Saccharibacteria	9.20E-05	8.04E-06	0	6.33E-07	5.12E-05	0.0004393	0.000512917	0.000165733	3.62E-05	0.000789775	3.02E-05	3.61E-05
p__Euryarchaeota	7.66E-05	0.0001311	0	0	0	0	0.00022285	0.000217017	4.31E-05	7.51E-05	8.90E-05	0
others	0.000205017	2.97E-06	0	3.33E-08	3.61E-05	1.14E-05	1.67E-08	0	-6.00E-08	0	-3.33E-08	5.57E-06

Supplementary Table S12. Quantity summary of differential enriched metabolites from Point 1 to Point 6

	P1	P2	P3	P4	P5	P6
Detected metabolites	25160	25042	24673	25272	25148	25136
Metabolites with name	1047	1045	1042	1047	1047	1048
Differential metabolites	518	442	646	1319	1272	806
Differential metabolites with name	30	15	26	45	50	43

Supplementary Table S13. 20 survival associated differential taxa

Item	DCB_fre	NDB_fre	DCB_mean	NDB_mean	wilcox.pvalue	BH-pvalue	FDR-pvalue	Enriched_group	LDA_score	LEfSe_pvalue
p__Fusobacteria	0.085	0.317	9.74E-05	0.001921056	0.005238702	0.039290269	0.039290269	NDB	3.036855752	0.005141265
c__CFGB3035	0.319	0.146	0.000570053	0.000127124	0.037386997	0.249246646	0.249246646	DCB	2.431935943	0.036878352
c__Fusobacteriia	0.085	0.317	9.74E-05	0.001921056	0.005238702	0.10477405	0.10477405	NDB	3.004468363	0.005141265
c__Bacilli	0.979	1	0.020364704	0.067864024	7.60E-05	0.006083373	0.006083373	NDB	4.386463023	7.47E-05
o__OFGB3035	0.319	0.146	0.000570053	0.000127124	0.037386997	0.269186378	0.269186378	DCB	2.315396638	0.036878352
o__Fusobacteriales	0.085	0.317	9.74E-05	0.001921056	0.005238702	0.141444967	0.141444967	NDB	3.025622052	0.005141265
o__Lactobacillales	0.957	1	0.019692304	0.061734366	3.34E-05	0.003607218	0.003607218	NDB	4.31946158	3.28E-05
f__FGB3035	0.319	0.146	0.000570053	0.000127124	0.037386997	0.270610644	0.270610644	DCB	2.455398218	0.036878352
f__Clostridiales_Family_XIII_Incertae_Sedis	0.362	0.561	0.000272379	0.000869827	0.02232908	0.231857149	0.231857149	NDB	2.579617765	0.022062371
f__Fusobacteriaceae	0.085	0.317	9.74E-05	0.00186661	0.005238702	0.132713797	0.132713797	NDB	2.995225234	0.005141265
g__Alistipes	0.957	0.854	0.086952694	0.046217273	0.032179221	0.498290357	0.498290357	DCB	4.286598894	0.031844172
g__GGB9615	0.681	0.415	0.004247255	0.002197244	0.023908403	0.475339049	0.475339049	DCB	3.030550162	0.023637223
g__Lacticaseibacillus	0.043	0.195	2.37E-05	0.000513602	0.024185549	0.475339049	0.475339049	NDB	2.432784865	0.02371232
g__Fusobacterium	0.085	0.317	9.74E-05	0.00186661	0.005238702	0.281177118	0.281177118	NDB	2.911359731	0.005141265
g__Streptococcus	0.83	1	0.014259055	0.025542085	0.001960739	0.125242219	0.125242219	NDB	3.783228299	0.00193325
g__Limosilactobacillus	0.404	0.732	0.001776996	0.0190409	0.000145078	0.048766495	0.048766495	NDB	3.951479529	0.000142537
s__Clostridium_sp_AF34_10BH	0.723	0.39	0.001740043	0.000427022	0.000796387	0.218807325	0.218807325	DCB	2.856789832	0.000783925
s__Streptococcus_anginosus	0.511	0.707	0.000482502	0.003659902	0.007019072	0.382413998	0.382413998	NDB	3.187304016	0.006928563
s__Bifidobacterium_dentium	0.383	0.61	0.001166983	0.008842222	0.004978221	0.382413998	0.382413998	NDB	3.619869795	0.004841233
s__Streptococcus_salivarius	0.787	0.902	0.010945813	0.014799329	0.021644098	0.517105725	0.517105725	NDB	3.689939643	0.021405934

Supplementary Table S14. Clinical characteristics of the study population in train and test sets.

Characteristic	Train set (n=36)			Test set (n=18)		
	No. of patients	DCB (%)	NDB (%)	No. of patients	DCB (%)	NDB (%)
Pathology						
ICC	20	7 (35.0)	13 (81.3)	14	6 (66.7)	8 (88.9)
GBC	8	7 (35.0)	1 (6.2)	1	0	1 (11.1)
ECC	8	6 (30.0)	2 (12.5)	3	3 (33.3)	0
Response						
Partial response	12	12 (60.0)	NA	4	4 (44.4)	NA
Stable disease	16	8 (40.0)	8 (50.0)	8	5 (55.6)	3 (33.3)
Progressive disease	8	NA	8 (50.0)	6	NA	6 (66.7)
Sex						
Female	13	8 (40.0)	5 (31.3)	7	4 (44.4)	3 (33.3)
Male	23	12 (60.0)	11 (68.7)	11	5 (55.6)	6 (66.7)
Age, y						
≥ 60	21	10 (50.0)	11 (68.7)	11	6 (66.7)	5 (55.6)
< 60	15	10 (50.0)	5 (31.3)	7	3 (33.3)	4 (44.4)
Hepatitis						
Yes	6	2 (10.0)	4 (25.0)	3	1 (11.1)	2 (22.2)
No	30	18 (90.0)	12 (75.0)	15	8 (88.9)	7 (77.8)
ECOG						
0	23	15 (75.0)	8 (50.0)	10	6 (66.7)	4 (44.4)
1	12	5 (25.0)	7 (43.8)	7	3 (33.3)	4 (44.4)
2	1	0	1 (6.2)	1	0	1 (11.1)
Child–Pugh						
A	28	17 (85.0)	11 (68.7)	13	7 (77.8)	6 (66.7)
B	8	3 (15.0)	5 (31.3)	5	2 (22.2)	3 (33.3)
Grade						
Poor	11	7 (35.0)	4 (25.0)	5	3 (33.3)	2 (22.2)
Moderate	14	8 (40.0)	6 (37.5)	7	4 (44.4)	3 (33.3)
Well	2	0	2 (12.5)	1	0	1 (11.1)
NA	9	5 (25.0)	4 (25.0)	5	2 (22.2)	3 (33.3)
TNM stage						
II	1	1 (5.0)	0	1	1 (11.1)	0
III	19	10 (50.0)	9 (56.3)	7	4 (44.4)	3 (33.3)
IV	16	9 (45.0)	7 (43.7)	10	4 (44.4)	6 (66.7)
Vascular invasion						
Yes	7	3 (15.0)	4 (25.0)	4	2 (22.2)	2 (22.2)
No	29	17 (85.0)	12 (75.0)	14	7 (77.8)	7 (77.8)
Intrahepatic metastasis						
Yes	28	17 (85.0)	11 (68.7)	13	7 (77.8)	6 (66.7)
No	8	3 (15.0)	5 (31.3)	5	2 (22.2)	3 (33.3)
Tumor number						

1	4	4 (20.0)	0	0	0	0
2	4	1 (5.0)	3 (18.8)	1	0	1 (11.1)
3	1	0	1 (6.2)	0	0	0
>3	16	11 (55.0)	5 (31.3)	10	7 (77.8)	3 (33.3)
NA	11	4 (20.0)	7 (43.7)	7	2 (22.2)	5 (55.6)
Size#						
≥ 5 cm	18	8 (40.0)	10 (62.5)	8	4 (44.4)	4 (44.4)
< 5 cm	18	12 (60.0)	6 (37.5)	10	5 (55.6)	5 (55.6)
Alpha fetoprotein						
≥ 20 U/mL	2	2 (10.0)	0	1	0	1 (11.1)
< 20 U/mL	33	17 (85.0)	16 (100.0)	16	9 (100.0)	7 (77.8)
NA	1	1 (5.0)	0	1	0	1 (11.1)
CA19-9						
≥ 200 U/mL	9	3 (15.0)	6 (37.5)	7	3 (33.3)	4 (44.4)
< 200 U/mL	27	17 (85.0)	10 (63.5)	11	6 (66.7)	5 (55.6)
Total bilirubin						
≥ 17 umol/L	16	10 (50.0)	6 (37.5)	8	3 (33.3)	5 (55.6)
< 17 umol/L	20	10 (50.0)	10 (63.5)	10	6 (66.7)	4 (44.4)
Direct bilirubin						
≥ 7 umol/L	14	6 (30.0)	8 (50.0)	3	3 (33.3)	0
< 7 umol/L	22	14 (70.0)	8 (50.0)	15	6 (66.7)	9 (100.0)
Bile acids						
≥ 10 umol/L	14	8 (40.0)	6 (37.5)	5	2 (22.2)	3 (33.3)
< 10 umol/L	19	9 (45.0)	10 (63.5)	13	7 (77.8)	6 (66.7)
NA	3	3 (15.0)	0	0	0	0
Therapeutic regimen						
IM+MTT	32	17 (85.0)	15 (93.8)	16	8 (88.9)	8 (88.9)
IM+MTT+CHEMO	4	3 (15.0)	1 (6.2)	2	1 (11.1)	1 (11.1)

#Maximum tumor diameter. DCB, durable clinical benefit; NDB, non-durable clinical benefit; ICC, intrahepatic cholangiocarcinoma; GBC, gallbladder cancer; ECC, extrahepatic cholangiocarcinoma; ECOG, Eastern Cooperative Oncology Group; TNM, tumor node metastasis; CA19-9, Carbohydrate antigen 19-9; IM, immunotherapy; MTT, molecular targeted therapy; CHEMO, chemotherapy.

Supplementary Table S15. The performances of all prediction models by Random Forest Analysis.

**Features**		
**Model 1** (microbiome)	**Model 2**(metabolome)	**Model 3**(microbiome + metabolome)
s__Anaeroglobus_geminatus<br>s__Streptococcus_anginosus<br>s__Olsenella_profusa<br>s__Alistipes_putredinis<br>s__Isoptericola_variabilis<br>s__Candidatus_Nanosynsacchari_sp_TM7_ANC_38_39_G1_1	4-[(Hydroxymethyl)nitrosoamino]-1-(3-pyridinyl)-1-butanone<br>Tryptamine<br>Pyrrolidine<br>4-(2,6,6-Trimethyl-1,3-cyclohexadien-1-yl)-2-butanone	s__Anaeroglobus_geminatus<br>s__Olsenella_profusa<br>4-[(Hydroxymethyl)nitrosoamino]-1-(3-pyridinyl)-1-butanone<br>s__Streptococcus_anginosus<br>Pyrrolidine
Train set:<br>AUC = 89.69% (95% CI :78.87%-100%)	Train set:<br>AUC = 86.25% (95% CI :74.38%-98.12%)	Train set:<br>AUC = 95.94% (95% CI :90.41%-100%)
Test set:<br>AUC = 72.22% (95% CI: 45.86%-98.58%)	Test set:<br>AUC = 75.31% (95% CI: 50.81%-99.81%)	Test set:<br>AUC = 83.95% (95% CI: 61.81%-100%)

Supplementary Table S16. Differential kegg pathways for metagenomic sequencing

Item	DCB_fre	NDB_fre	DCB_mean	NDB_mean	wilcox.pvalue	BH-pvalue	FDR-pvalue	Enriched_group	LDA_score	LEfSe_pvalue
ko00020:Citrate cycle (TCA cycle)	0.979	0.976	0.014229438	0.011586999	0.007527481	0.217928265	0.217928265	DCB	3.17610888	0.007434167
ko00600:Sphingolipid metabolism	0.915	0.756	0.006379065	0.004435629	0.039219957	0.439676356	0.439676356	DCB	2.98214803	0.038822583
ko00630:Glyoxylate and dicarboxylate metabolism	1	1	0.011178131	0.009968329	0.006895996	0.217928265	0.217928265	DCB	2.870111188	0.006809744
ko02048:Prokaryotic defense system	1	1	0.006820711	0.005623393	0.046500819	0.459193691	0.459193691	DCB	2.858410926	0.046042757
ko00051:Fructose and mannose metabolism	1	1	0.010895851	0.009712205	0.022395862	0.31902622	0.31902622	DCB	2.79835793	0.022151001
ko00650:Butanoate metabolism	1	1	0.006836289	0.005909694	0.047428456	0.459193691	0.459193691	DCB	2.795768	0.046962711
ko00195:Photosynthesis	0.106	0	0.001396203	0	0.033452775	0.419143591	0.419143591	DCB	2.788735122	0.03259545
ko00541:O-Antigen nucleotide sugar biosynthesis	0.979	0.976	0.005568622	0.004580192	0.017719553	0.31902622	0.31902622	DCB	2.757267953	0.017520003
ko00680:Methane metabolism	0.936	0.927	0.006736464	0.005927476	0.004165087	0.217928265	0.217928265	DCB	2.664875727	0.00411039
ko00260:Glycine, serine and threonine metabolism	1	1	0.013256977	0.012479112	0.045588501	0.459193691	0.459193691	DCB	2.644306323	0.045138027
ko00190:Oxidative phosphorylation	1	1	0.008396517	0.007680165	0.007071449	0.217928265	0.217928265	DCB	2.605523147	0.006983228
ko00998:Biosynthesis of various antibiotics	0.915	0.854	0.002075595	0.001399512	0.019959875	0.31902622	0.31902622	DCB	2.566792724	0.019738246
ko03051:Proteasome	0.702	0.829	0.000326334	0.000624178	0.022466635	0.31902622	0.31902622	NDB	2.444576291	0.022219427
ko03009:Ribosome biogenesis	1	1	0.006023241	0.006408655	0.019173537	0.31902622	0.31902622	NDB	2.455231369	0.018959716
ko01002:Peptidases and inhibitors	1	1	0.00217992	0.002378499	0.027811269	0.370237522	0.370237522	NDB	2.506083805	0.027515854
ko00627:Aminobenzoate degradation	0.83	0.927	0.000646542	0.0010073	0.010403926	0.246226254	0.246226254	NDB	2.535890911	0.010279183
ko00760:Nicotinate and nicotinamide metabolism	1	1	0.006363157	0.006893063	0.005627288	0.217928265	0.217928265	NDB	2.620465727	0.005555474
ko02010:ABC transporters	0.894	0.951	0.004910835	0.00592759	0.035721911	0.422709278	0.422709278	NDB	2.752265737	0.03535558
ko00230:Purine metabolism	1	1	0.010942973	0.01194609	0.005484487	0.217928265	0.217928265	NDB	2.785608587	0.00541432

ko02024:Quorum sensing	0.809	0.878	0.004559577	0.005687844	0.020024323	0.31902622	0.31902622	NDB	2.786303663	0.019801786
ko00552:Teichoic acid biosynthesis	0.957	0.951	0.00410935	0.005526724	0.001137745	0.217928265	0.217928265	NDB	2.877803792	0.001121107
ko00194:Photosynthesis proteins	0.574	0.805	0.003023476	0.00471478	0.008185099	0.217928265	0.217928265	NDB	2.961995177	0.008082859

Supplementary Table S17. Differential metacyc pathways for metagenomic sequencing

Item	DCB_fre	NDB_fre	DCB_mean	NDB_mean	wilcox.pvalue	BH-pvalue	FDR-pvalue	Enriched_group	LDA_score	LEfSe_pvalue
PANTO-PWY: phosphopantothenate biosynthesis I	1	1	0.006420781	0.005119213	0.00841603	0.23186163	0.23186163	DCB	2.836695212	0.008312902
PANTOSYN-PWY: pantothenate and coenzyme A biosynthesis I	1	1	0.006574306	0.005413787	0.034320108	0.366857283	0.366857283	DCB	2.793839865	0.033966185
HISDEG-PWY: L-histidine degradation I	1	1	0.003805878	0.002689273	0.020961314	0.308502995	0.308502995	DCB	2.768764174	0.020730176
PWY-6703: preQ0 biosynthesis	1	1	0.004761182	0.00376406	0.040425731	0.390782063	0.390782063	DCB	2.757857755	0.040018818
PWY-5030: L-histidine degradation III	1	1	0.003124531	0.001952181	0.007250901	0.23186163	0.23186163	DCB	2.75771043	0.00716067
THISYN-PWY: superpathway of thiamin diphosphate biosynthesis I	1	1	0.005110072	0.004070651	0.005484487	0.23186163	0.23186163	DCB	2.743624116	0.00541432
1CMET2-PWY: N10-formyl-tetrahydrofolate biosynthesis	1	1	0.00766039	0.006824214	0.041250087	0.391875828	0.391875828	DCB	2.668587124	0.040836146
PWY-7663: gondoate biosynthesis (anaerobic)	1	1	0.006425854	0.005709712	0.006232637	0.23186163	0.23186163	DCB	2.634827044	0.00615389
ARGININE-SYN4-PWY: L-ornithine de novo biosynthesis	1	1	0.003154731	0.002288602	0.027223149	0.357141786	0.357141786	DCB	2.615958852	0.026933135
NAGLIPASYN-PWY: lipid IVA biosynthesis	1	1	0.002604236	0.001858626	0.006895996	0.23186163	0.23186163	DCB	2.576743753	0.006809744
ASPASN-PWY: superpathway of L-aspartate and L-asparagine biosynthesis	1	1	0.004848145	0.00417382	0.012092434	0.3028605	0.3028605	DCB	2.560549939	0.011949987
PWY-6892: thiazole biosynthesis I (E. coli)	1	1	0.004674	0.004173	0.042941	0.392488	0.392488	DCB	2.343244	0.0425132

			412	834	569	5	5		312	95
PWY-7332: superpathway of UDP-N-acetylglucosamine-derived O-antigen building blocks biosynthesis	0.894	0.878	0.000674<br>764	0.000410<br>168	0.044981<br>987	0.392488<br>5	0.392488<br>5	DCB	2.142271<br>089	0.0445362<br>53
PWY-5508: adenosylcobalamin biosynthesis from cobyrinate a,c-diamide II	0	0.098	0	7.10E-06	0.030240<br>563	0.362229<br>356	0.362229<br>356	NDB	2.039177<br>105	0.0293678<br>68
PWY-5430: meta cleavage pathway of aromatic compounds	0.021	0.146	5.06E-07	4.46E-06	0.032419<br>389	0.365599<br>887	0.365599<br>887	NDB	2.054348<br>591	0.0317046<br>69
LACTOSECAT-PWY: lactose and galactose degradation I	1	0.976	0.000314<br>752	0.000525<br>356	0.035767<br>482	0.371846<br>843	0.371846<br>843	NDB	2.059906<br>746	0.0354008<br>43
PWY-6138: CMP-N-acetylneuraminate biosynthesis I (eukaryotes)	0.043	0.22	2.53E-06	7.98E-06	0.017639<br>349	0.308502<br>995	0.308502<br>995	NDB	2.063096<br>208	0.0172946<br>82
PWY-7003: glycerol degradation to butanol	0.915	0.976	0.000529<br>502	0.000722<br>548	0.029006<br>289	0.362229<br>356	0.362229<br>356	NDB	2.089968<br>442	0.0286999<br>32
ARGORNPROST-PWY: arginine, ornithine and proline interconversion	0.957	0.976	0.000195<br>301	0.000429<br>286	0.000161<br>565	0.044511<br>255	0.044511<br>255	NDB	2.090876<br>951	0.0001588<br>77
PWY-5182: toluene degradation II (aerobic) (via 4-methylcatechol)	0.234	0.512	0.000230<br>593	0.000478	0.007588<br>952	0.231861<br>63	0.231861<br>63	NDB	2.168853<br>336	0.0074799<br>53
PWY-5265: peptidoglycan biosynthesis II (staphylococci)	0.553	0.756	0.000155<br>793	0.000391<br>861	0.034621<br>74	0.366857<br>283	0.366857<br>283	NDB	2.176514<br>883	0.0342571<br>43
PWY-5180: toluene degradation I (aerobic) (via o-cresol)	0.234	0.512	0.000230<br>593	0.000478	0.007588<br>952	0.231861<br>63	0.231861<br>63	NDB	2.182196<br>259	0.0074799<br>53
PWY-6595: superpathway of guanosine nucleotides degradation (plants)	0.915	0.976	0.000278<br>696	0.000550<br>244	0.013914<br>494	0.306675<br>45	0.306675<br>45	NDB	2.185295<br>19	0.0137531<br>86
PWY-6549: L-glutamine biosynthesis III	1	1	0.000952<br>994	0.001250<br>765	0.019173<br>537	0.308502<br>995	0.308502<br>995	NDB	2.193010<br>418	0.0189597<br>16
P185-PWY: formaldehyde assimilation III (dihydroxyacetone cycle)	1	1	0.000613	0.000847	0.042088	0.392488	0.392488	NDB	2.220590	0.0416675

			57	446	638	5	5		149	77
PWY-7198: pyrimidine deoxyribonucleotides de novo biosynthesis IV	1	1	0.001756589	0.002236859	0.022395862	0.308502995	0.308502995	NDB	2.358191913	0.022151001
PWY-5121: superpathway of geranylgeranyl diphosphate biosynthesis II (via MEP)	0.894	0.951	0.00060507	0.001020562	0.01357868	0.30667545	0.30667545	NDB	2.368703168	0.013420794
PWY-622: starch biosynthesis	0.66	0.805	0.000556115	0.001048511	0.032512513	0.365599887	0.365599887	NDB	2.410362813	0.032171156
PWY-6901: superpathway of glucose and xylose degradation	1	1	0.003036791	0.00351092	0.020961314	0.308502995	0.308502995	NDB	2.433147322	0.020730176
PENTOSE-P-PWY: pentose phosphate pathway	1	1	0.003345842	0.003949228	0.028410312	0.362229356	0.362229356	NDB	2.484869203	0.028109415
PWY-7560: methylerythritol phosphate pathway II	0.894	0.951	0.000884763	0.001505403	0.015250047	0.308502995	0.308502995	NDB	2.513604574	0.015075124
PWY-6270: isoprene biosynthesis I	0.894	0.951	0.000833456	0.001461223	0.018718796	0.308502995	0.308502995	NDB	2.525180808	0.018509369
PWY-6527: stachyose degradation	1	1	0.003599956	0.004169432	0.045588501	0.3924885	0.3924885	NDB	2.526825004	0.045138027
NONOXIPENT-PWY: pentose phosphate pathway (non-oxidative branch)	1	1	0.006022162	0.006662556	0.049330457	0.408097966	0.408097966	NDB	2.533281526	0.048849056
P124-PWY: Bifidobacterium shunt	0.766	0.854	0.00054169	0.001259965	0.017511669	0.308502995	0.308502995	NDB	2.564950616	0.017313466
METSYN-PWY: L-homoserine and L-methionine biosynthesis	1	1	0.003198867	0.003871853	0.039615385	0.389787094	0.389787094	NDB	2.57359741	0.03921541
PWY0-781: aspartate superpathway	1	1	0.001976888	0.002697779	0.017127296	0.308502995	0.308502995	NDB	2.595906379	0.016933613
MET-SAM-PWY: superpathway of S-adenosyl-L-methionine biosynthesis	1	1	0.003243	0.003978	0.020501	0.308502	0.308502	NDB	2.610870	0.0202745

			114	214	262	995	995		841	56
PWY66-400: glycolysis VI (metazoan)	1	1	0.004071<br>177	0.004883<br>171	0.029641<br>809	0.362229<br>356	0.362229<br>356	NDB	2.617789<br>792	0.0293297<br>08
PYRIDNUCSAL-PWY: NAD salvage pathway I	0.979	0.976	0.001605<br>966	0.002400<br>527	0.008312<br>617	0.231861<br>63	0.231861<br>63	NDB	2.621794<br>552	0.0082106<br>24
NONMEVIPP-PWY: methylerythritol phosphate pathway I	0.894	0.951	0.001442<br>46	0.002320<br>242	0.014560<br>913	0.308502<br>995	0.308502<br>995	NDB	2.666459<br>011	0.0143929<br>79
PWY-6471: peptidoglycan biosynthesis IV (Enterococcus faecium)	1	0.951	0.001437<br>87	0.002226<br>051	0.026078<br>385	0.350468<br>053	0.350468<br>053	NDB	2.669848<br>777	0.0257989<br>44
PWY-2941: L-lysine biosynthesis II	1	1	0.001886<br>978	0.003048<br>469	0.001396<br>625	0.096192<br>521	0.096192<br>521	NDB	2.723441<br>082	0.0013765<br>18
OANTIGEN-PWY: O-antigen building blocks biosynthesis (E. coli)	1	1	0.003379<br>6	0.004460<br>783	0.000772	0.060767<br>44	0.060767<br>44	NDB	2.730931<br>799	0.0007603<br>87
UDPNAGSYN-PWY: UDP-N-acetyl-D-glucosamine biosynthesis I	1	1	0.002986<br>43	0.004244<br>664	0.000442<br>728	0.044568<br>589	0.044568<br>589	NDB	2.807096<br>823	0.0004358<br>1

Supplementary Table S18. Enrichment metabolic pathways for differential metabolite annotation

Pathway	Description	CpdRatio	BgRatio	pvalue	p.adjust	Compound ID
hsa00380	Tryptophan metabolism	2/5	13/276	0.0189480269762907	0.1071203946799	C00398/C10164
hsa00980	Metabolism of xenobiotics by cytochrome P450	1/5	3/276	0.0535601973399498	0.1071203946799	C19563
hsa05204	Chemical carcinogenesis - DNA adducts	1/5	3/276	0.0535601973399498	0.1071203946799	C19563
hsa00260	Glycine, serine and threonine metabolism	1/5	15/276	0.245355243306174	0.301858239084517	C06231
hsa00240	Pyrimidine metabolism	1/5	17/276	0.274046423268892	0.301858239084517	C00178
hsa04080	Neuroactive ligand-receptor interaction	1/5	19/276	0.301858239084517	0.301858239084517	C00398

Supplementary Table S19. The contribution of different clinical factors to the analysis of CCA based on species

tax	CCA1	CCA2	R2	pvalue
Tsize	-0.339016834	-0.940780307	0.111883463	0.0716
Dbil	0.971469622	0.237164022	0.416194618	0.0019
Tbil	0.972440883	0.233149587	0.446192979	0.001
Sex	-0.862065892	-0.50679621	0.057189685	0.2965
Age	0.402024728	-0.91562881	0.103338454	0.0969
TNM	-0.783118029	-0.6218731	0.029025287	0.5423
Response	0.446911979	-0.894577936	0.075715174	0.1793
ChildPugh	0.83723051	-0.546850138	0.22707951	0.0036
Ecog	0.65917563	-0.751989022	0.273546019	0.0012
Hepatitis	-0.194621799	-0.980878359	0.316477238	4.00E-04
CA199	0.998521117	-0.054365236	0.039078958	0.1993
IHM	0.418743775	-0.908104427	0.084030933	0.1468
VI	-0.407152187	-0.913360333	0.245712846	0.0018
BA	0.923309947	-0.384055649	0.12290841	0.0601
AFP	0.566528258	0.824042313	0.003798911	0.761
Treatment	0.343193943	-0.939264562	0.029696785	0.4494

Supplementary Table S20. The contribution of different clinical factors to the analysis of CCA based on 20 survival differential taxa

tax	CCA1	CCA2	R2	pvalue
Tsize	-0.926980809	-0.375108757	0.015614261	0.6296
Dbil	-0.999771091	-0.021395478	0.121631514	0.0371
Tbil	-0.999740949	-0.022760361	0.138300723	0.0312
Sex	0.433066496	0.901361975	0.003970429	0.9209
Age	-0.984472314	0.175539918	0.101951622	0.0535
TNM	0.661569252	-0.749884074	0.017957177	0.5952
Response	-0.986238878	-0.165326571	0.084366977	0.077
ChildPugh	-0.998860774	0.047719535	0.121290621	0.0343
Ecog	-0.986063602	0.166368788	0.132818124	0.0244
Hepatitis	0.040075124	-0.99919667	0.188376793	0.0058
CA199	-0.965468636	-0.260519313	0.004452359	0.7598
IHM	-0.957568614	-0.288205395	0.049649165	0.2239
VI	0.993681396	0.112237618	0.001507321	0.9571
BA	-0.956003529	-0.293355163	0.134865205	0.0313
AFP	-0.999629059	0.027235002	0.000376561	0.9727
Treatment	-0.934179559	0.35680324	0.038528496	0.31

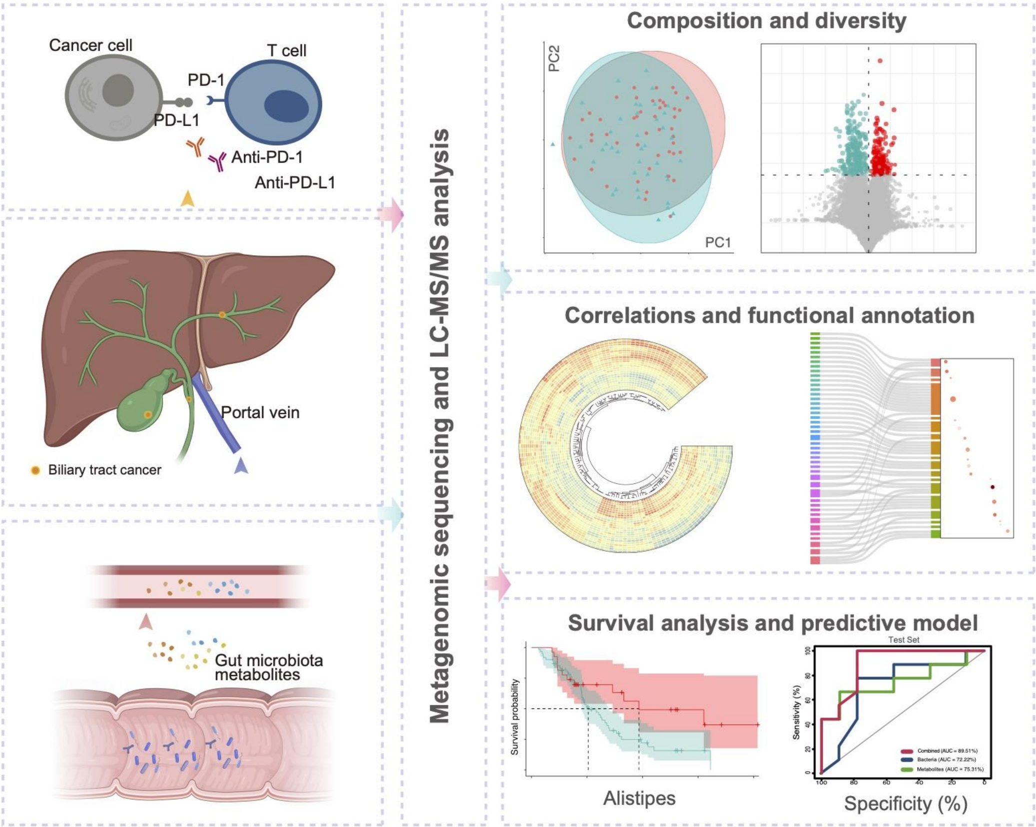

Supplementary Fig. S1. Study workflow

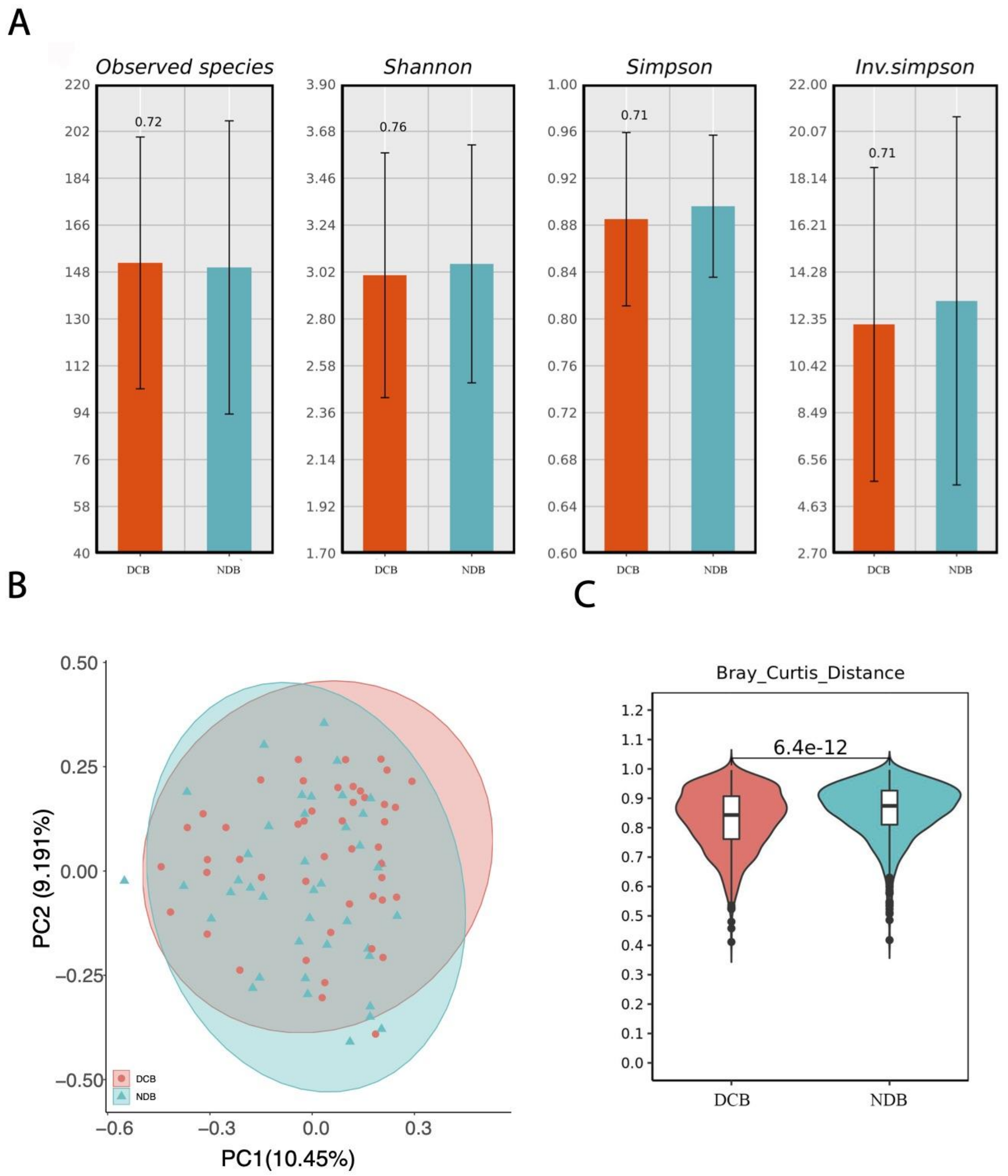

Supplementary Fig. S2. The alpha diversity and beta diversity between DCB and NDB group based on species.

(A)Alpha diversity. (B)Principal coordinate analysis (PCoA) of beta diversity measurements by Bray-Curtis distances. ANOSIM, R=0.04, P=0.025. (C)Beta diversity.

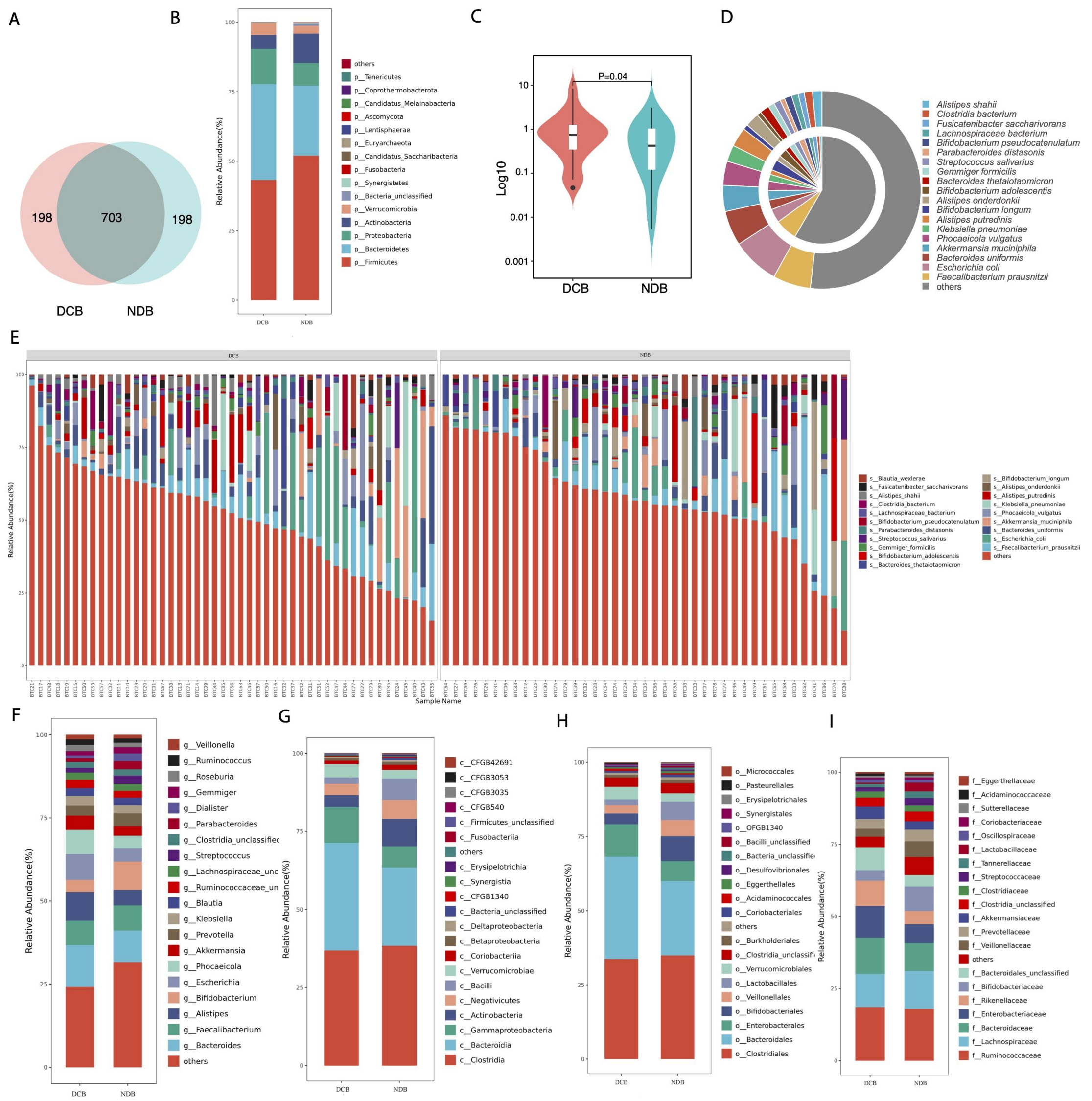

Supplementary Fig. S3. Gut microbiome composition in the DCB and NDB group.

(A)Venn diagram of common and unique species. (B)Gut microbiome composition at the phylum level. (C)*Bacteroidetes*/*Firmicutes* ratio (B/F). (D)Gut microbiome composition at the species level. The inner circle shows the species belonging to NDB, and the outer circle shows the species belonging to DCB. (E)The composition of the top 20 species of each baseline sample. Gut microbiome composition at the genus (F), class (G), order (H), and family (I) level.

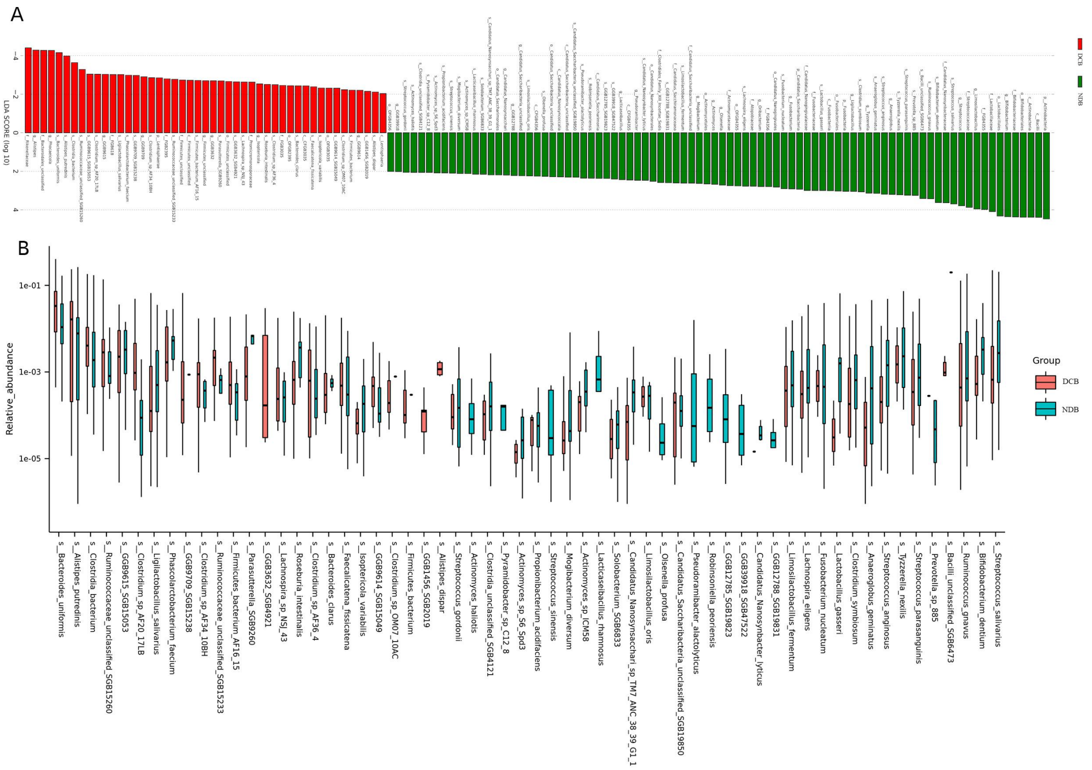

Supplementary Fig. S4. Significantly different abundant taxa in the DCB group and NDB.

(A)All enriched taxa in DCB or NDB group. (B)Relative expression abundances in the DCB and NDB group based on species.

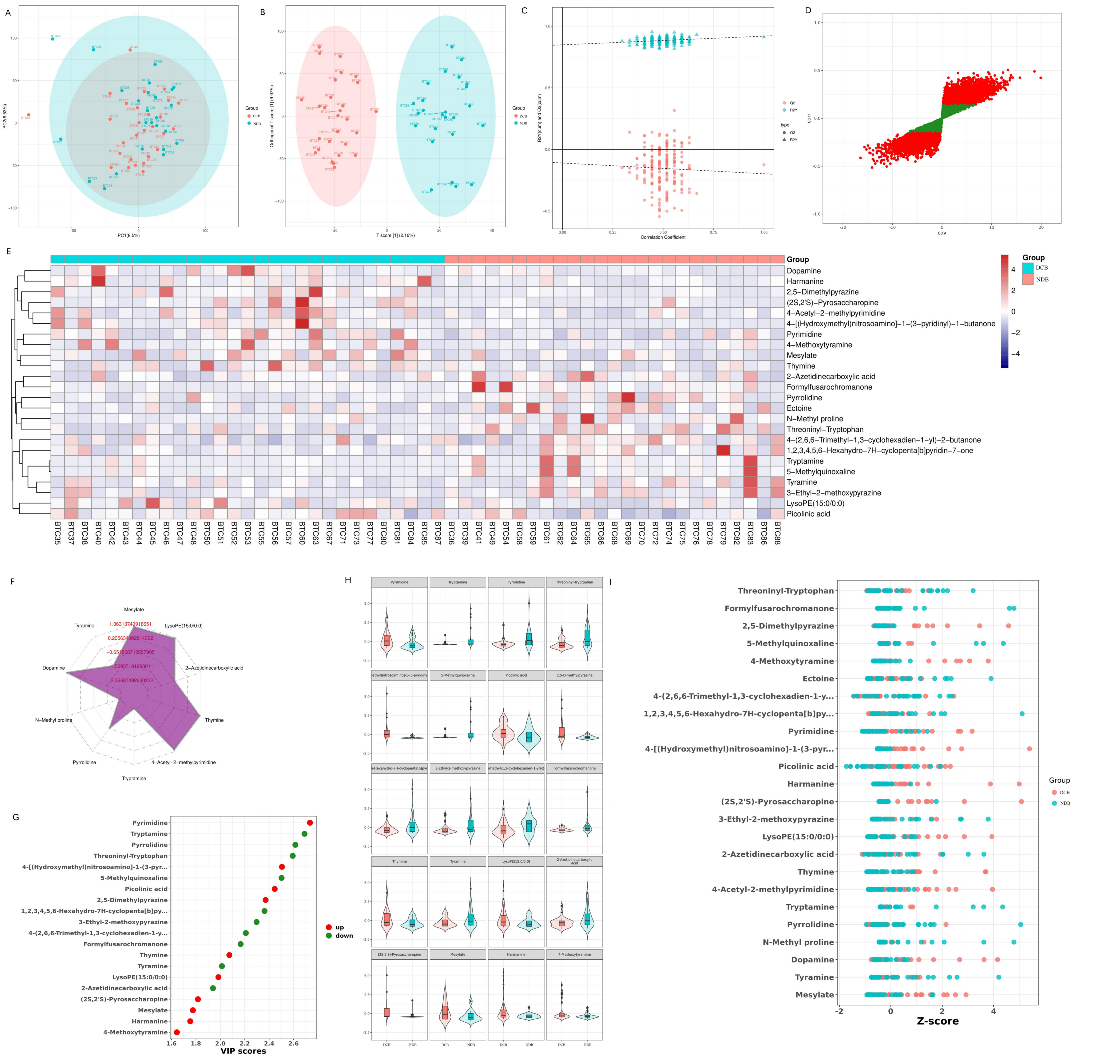

Supplementary Fig. S5. Differential metabolites for group DCB vs NDB.

(A)Score scatter plot of PCA. (B)Score scatter plot of OPLS-DA model. (C)Permutation test of OPLS-DA model. (D)OPLS-DA S-plot. (E)Heatmap of hierarchical clustering analysis. (F)Radar chart analysis. (G)VIP scores. (H)Violin Plot of differential metabolites. (I)Z-score of differential metabolites.

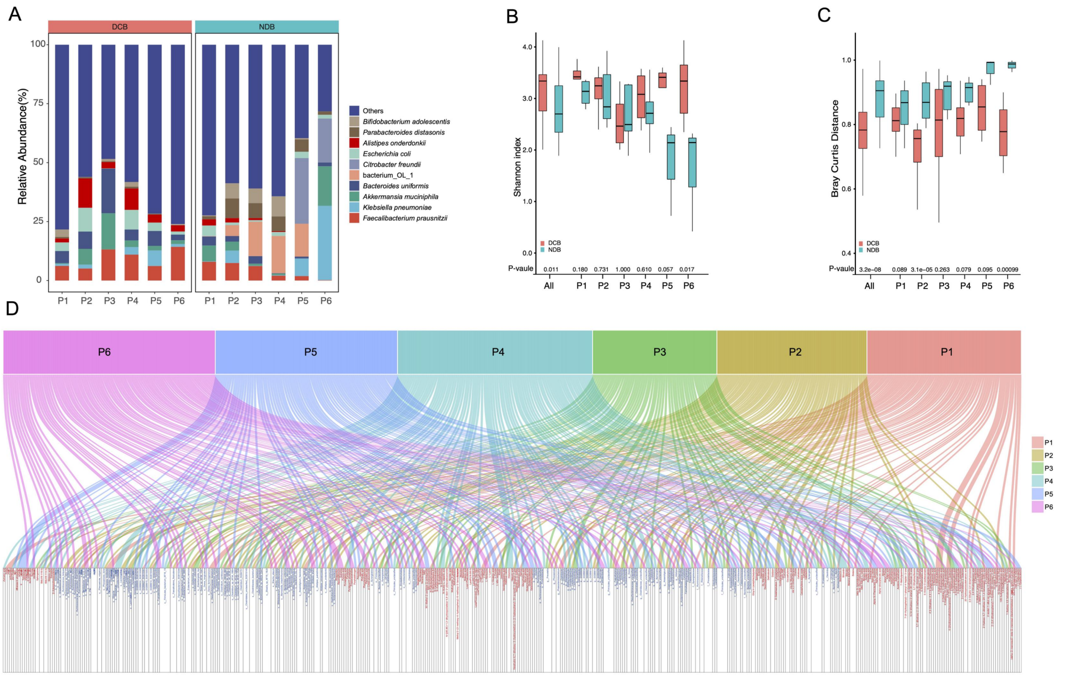

Supplementary Fig. S6. Dynamic point analysis in DCB and NDB groups.

(A)Dynamic microbial composition at the species level. (B) Dynamic point alpha diversity based on species_shannon. (C) Dynamic point beta diversity based on species_Bray-Curtis distances. (D) Differential taxa and metabolites from Point 1 to Point 6. Blue font represents differential taxa, and red represents differential metabolites.

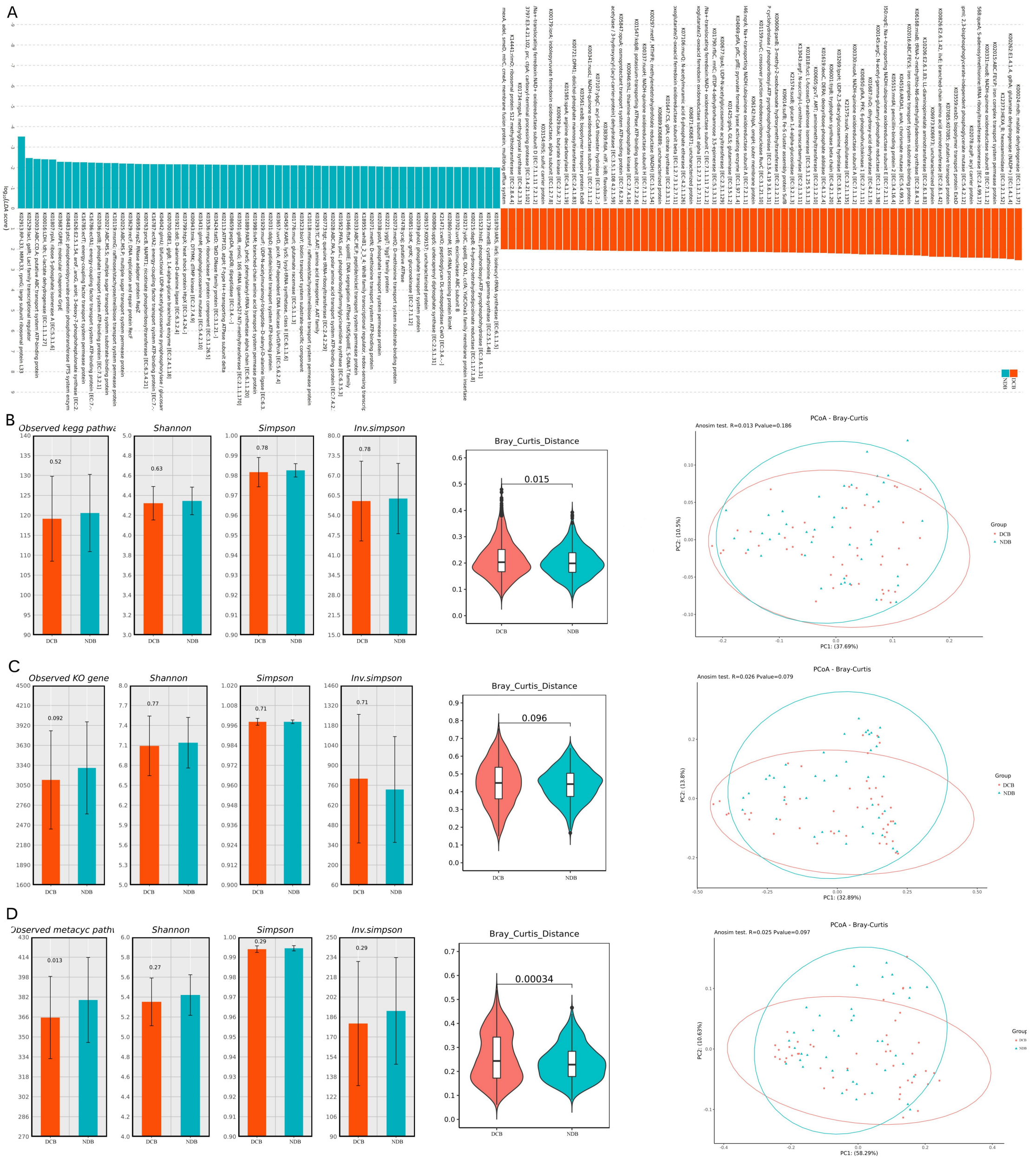

Supplementary Fig. S7. Functional annotation of metagenomic and metabolomics.

(A)Significantly different KO genes in the DCB and NDB group. The alpha diversity and beta diversity between DCB and NDB group based on Kegg pathway (B), ko gene (C), and metabolic pathway (D).

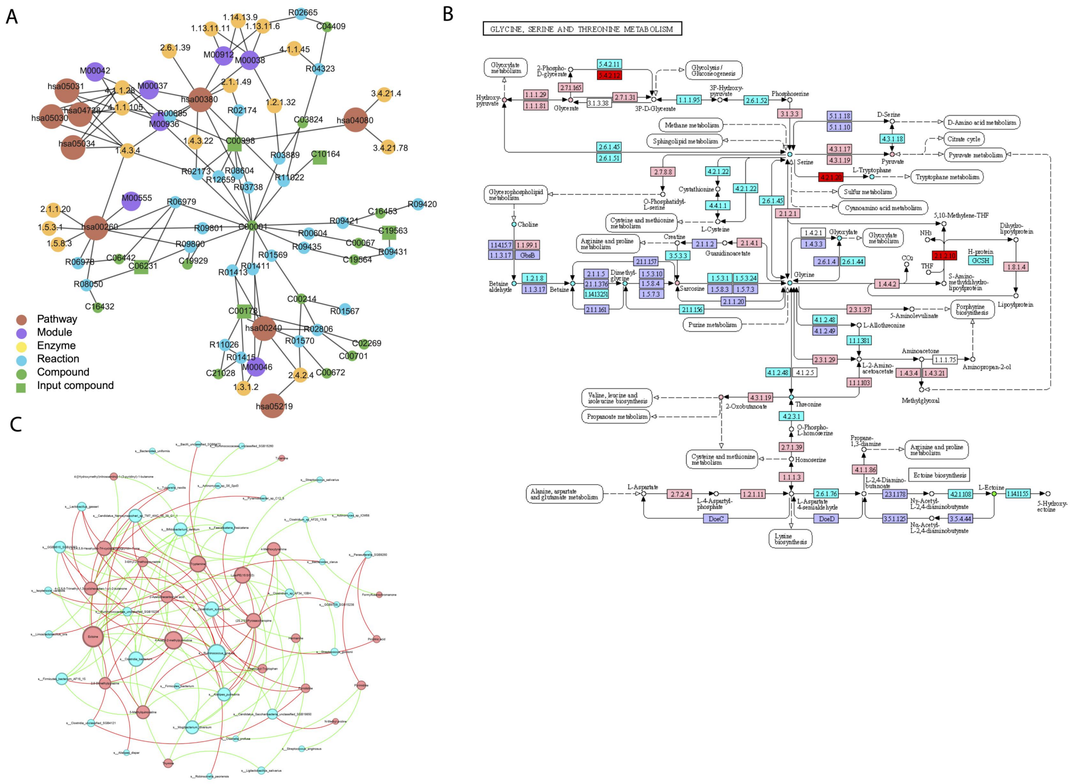

Supplementary Fig. S8. Correlations between microbes and metabolites.

(A) Cooccurrence network of enrichment metabolic pathways by LC-MS/MS analysis. (B) KO00260 pathway co-plotted the differential KO gene and metabolites. Red indicates that the DCB group is significantly higher than the NDB group; green indicates that it is significantly lower in the DCB group than in the NDB group; pink indicates that it was higher in the DCB group than in the NDB group but not significant; bright green indicates that the DCB group is lower than the NDB group but not significant. (C)Correlations analysis of all differential species and differential metabolites.

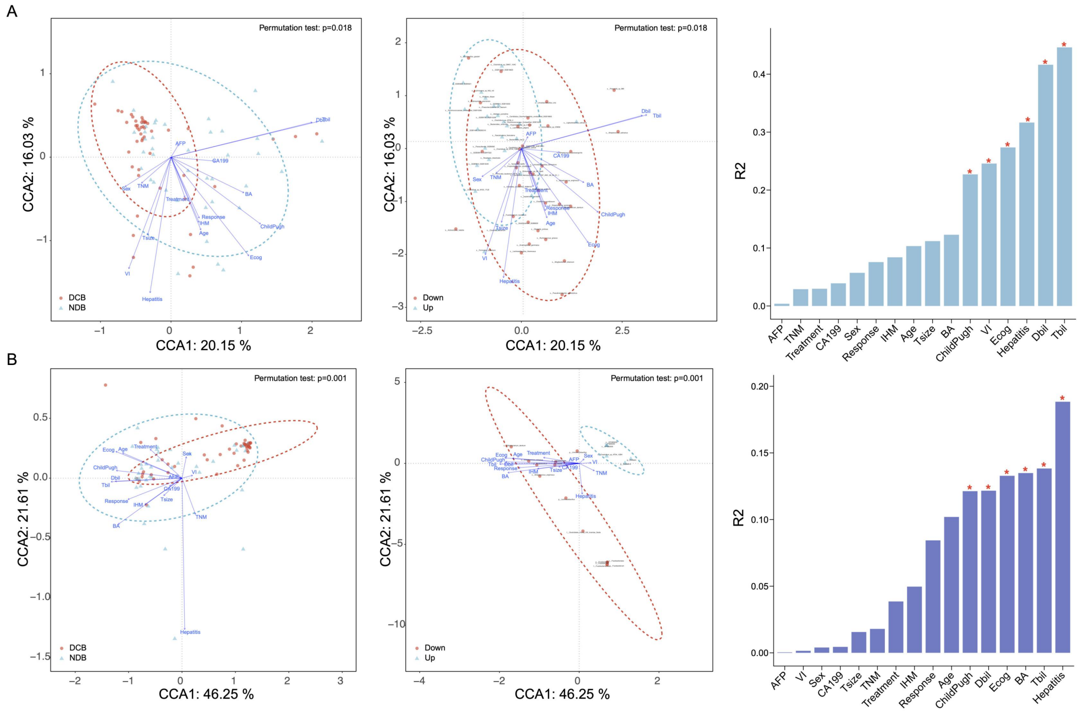

Supplementary Fig. S9. Gut microbiome distribution was affected by clinical factors.

Canonical correspondence analysis (CCA) with permutation test showed the clinical factors associated with the distribution of patients with BTC, the different species enriched in the DCB and NDB group, and contribution of different clinical factors to CCA for all differential taxa (A) and survival differential taxa (B). *P<0.05.

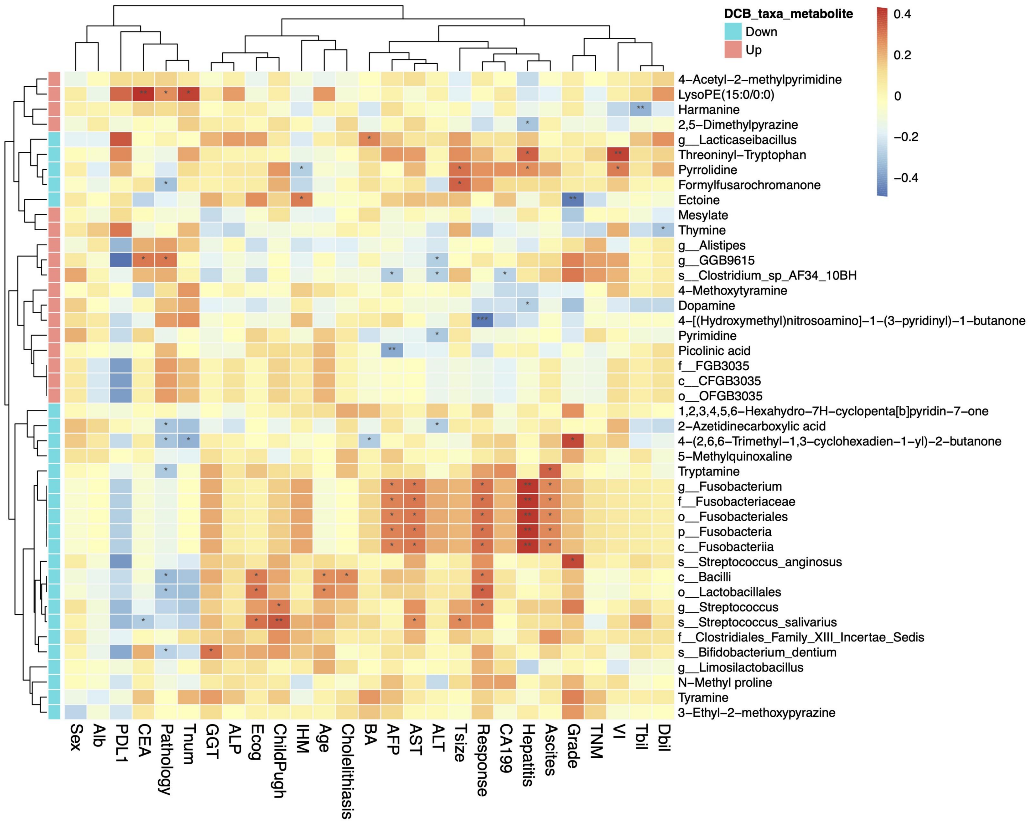

Supplementary Fig. S10. Correlations of clinical factors with differential metabolites and survival associated differentially taxa (Spearman's rank correlation with two-tailed P values). *P < 0.05; **P < 0.01; ***P < 0.001.

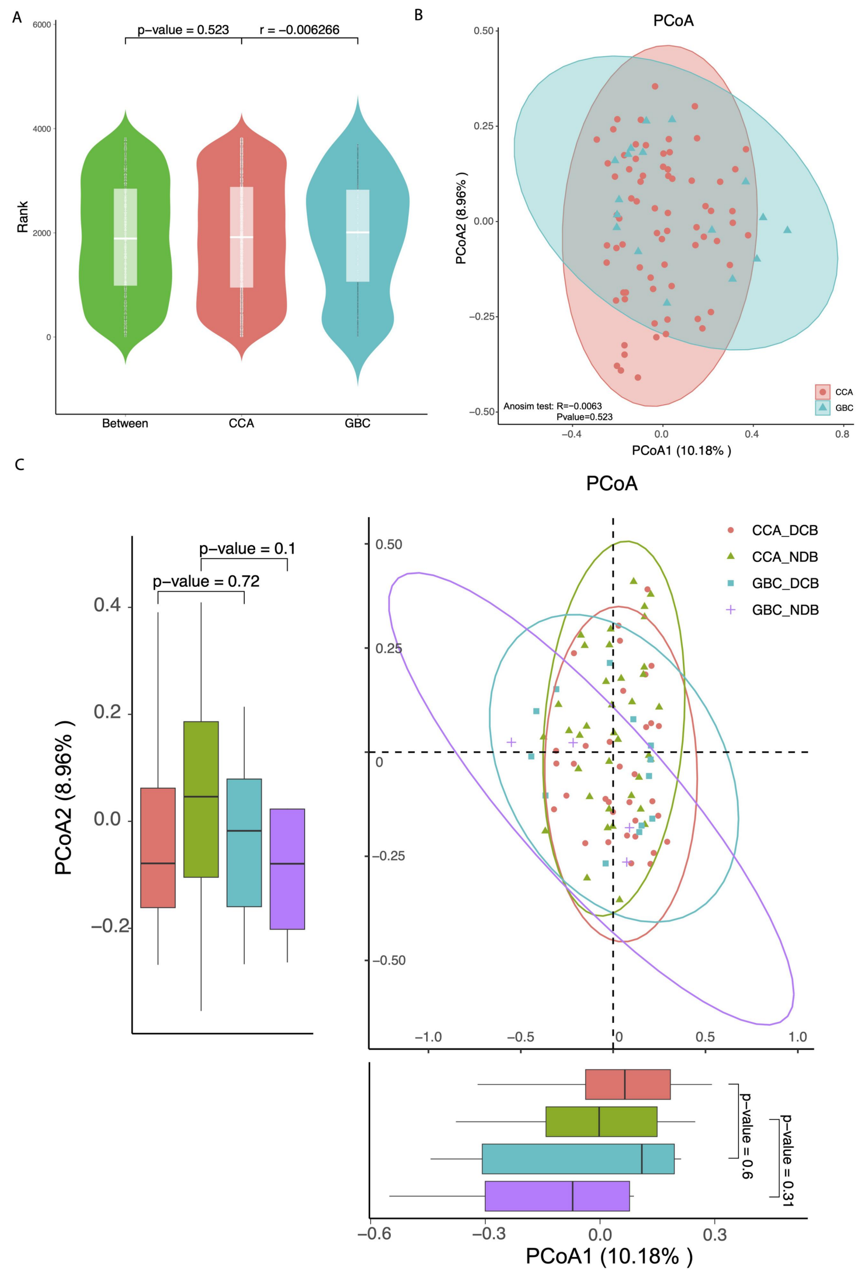

Supplementary Fig. S11. Comparison of intergroup differences between cholangiocarcinoma and gallbladder cancer.

(A-B)Principal Coordinate analysis (PCoA) based on Bray-Curtis distances between cholangiocarcinoma (CCA) and gallbladder cancer (GBC) groups. (C) Comparison of PCoA between the four groups (CCA_DCB, CCA_NDB, GBC_DCB, and GBC_NDB).

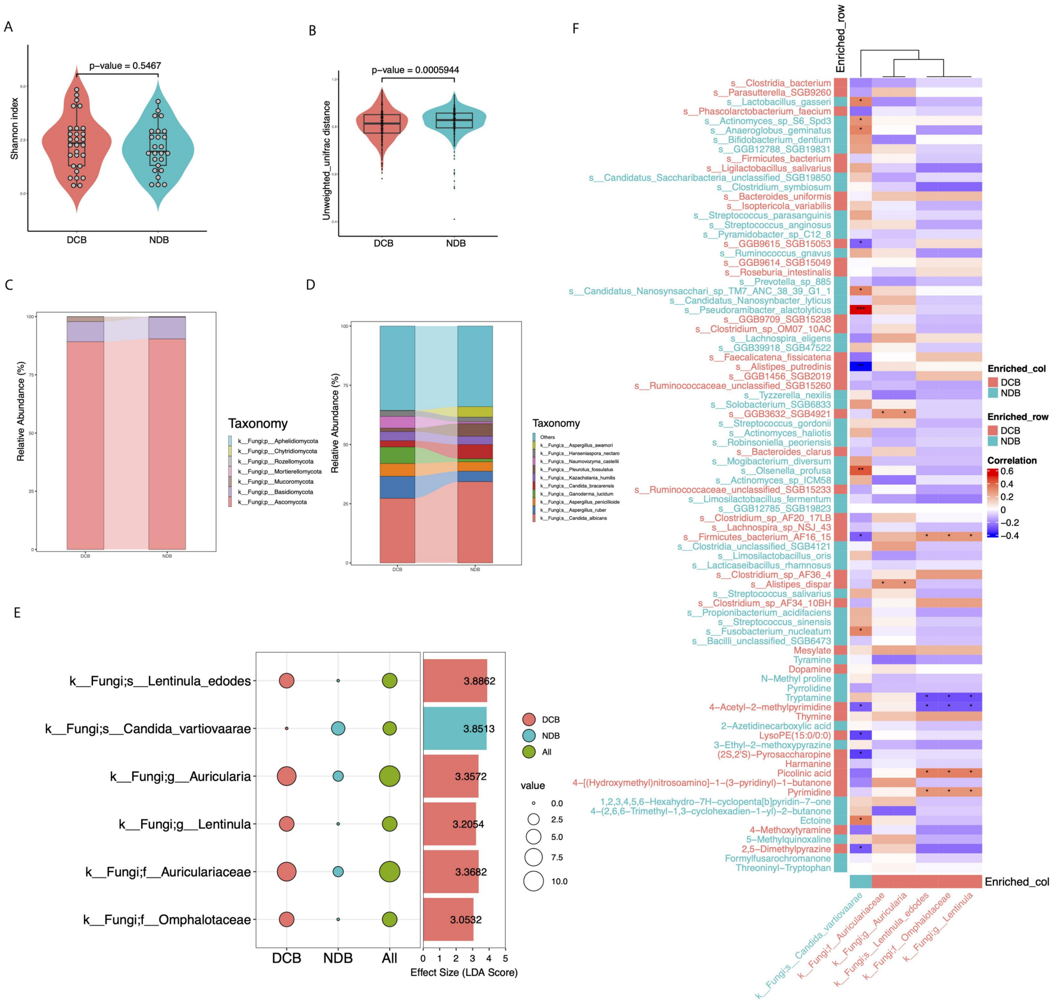

Supplementary Fig. S12. The differences between the DCB and NDB groups based on gut fungi.

(A) alpha diversity. (B) Beta diversity. (C) Significantly different fungi. (D) Association analysis of differential fungi with differential bacteria and metabolites.
